# Numerical Modeling of Sub-Wavelength Anti-Reflective Structures for Solar Module Applications

**DOI:** 10.3390/nano4010087

**Published:** 2014-01-29

**Authors:** Katherine Han, Chih-Hung Chang

**Affiliations:** School of Chemical, Biological, and Environmental Engineering, Oregon State University, Corvallis, OR 97331, USA; E-Mail: chih-hung.chang@oregonstate.edu

**Keywords:** optical modeling, antireflective, sub-wavelength, FDTD, RCWA, effective medium theory

## Abstract

This paper reviews the current progress in mathematical modeling of anti-reflective subwavelength structures. Methods covered include effective medium theory (EMT), finite-difference time-domain (FDTD), transfer matrix method (TMM), the Fourier modal method (FMM)/rigorous coupled-wave analysis (RCWA) and the finite element method (FEM). Time-based solutions to Maxwell’s equations, such as FDTD, have the benefits of calculating reflectance for multiple wavelengths of light per simulation, but are computationally intensive. Space-discretized methods such as FDTD and FEM output field strength results over the whole geometry and are capable of modeling arbitrary shapes. Frequency-based solutions such as RCWA/FMM and FEM model one wavelength per simulation and are thus able to handle dispersion for regular geometries. Analytical approaches such as TMM are appropriate for very simple thin films. Initial disadvantages such as neglect of dispersion (FDTD), inaccuracy in TM polarization (RCWA), inability to model aperiodic gratings (RCWA), and inaccuracy with metallic materials (FDTD) have been overcome by most modern software. All rigorous numerical methods have accurately predicted the broadband reflection of ideal, graded-index anti-reflective subwavelength structures; ideal structures are tapered nanostructures with periods smaller than the wavelengths of light of interest and lengths that are at least a large portion of the wavelengths considered.

## 1. Introduction

Recent trends of global climate change and impending petroleum shortages have encouraged researchers to develop a variety of renewable energy production methods, solar electricity generation being among the most popular of solutions. Commercially available monocrystalline silicon solar cell efficiency is currently above 24%, a mere 5% below the theoretical maximum. As solar cell production becomes cheaper, the cost of installed solar modules is beginning to depend more on module production and installation. One way to reduce the cost of installed solar panels is to minimize losses due to light reflection at interfaces, including but not limited to the air/glass and adhesive/silicon interfaces. This paper addresses the current status of mathematical modeling of anti-reflective sub-wavelength structures (ARSWS), provides the background on the most popular of modeling techniques for ARSWS, and suggests appropriate applications for each technique.

### 1.1. Scope

This paper is intended to be a review of the most commonly used methods for optical modeling of anti-reflective subwavelength structures. Optical modeling methods have developed over time and, with the introduction of advanced computing resources, have largely discarded methods that include non-rigorous assumptions. Likewise, with the wide variety of optical modeling methods available, some methods have become more popular than others due to reasons other than their computational ability, such as availability of commercial software or abundant use in the literature. Currently, only four major modeling methods are commonly used in the field of ARSWS: finite-difference time-domain (FDTD), finite element method (FEM), transfer matrix method (TMM), and rigorous coupled-wave analysis or Fourier modal method (RCWA/FMM). Of those, all but TMM are capable of describing the geometry of subwavelength structures; TMM relies on an effective media approximation for more complicated geometry. The mathematical approach for each of these methods is different, resulting in different advantages and disadvantages in modeling capabilities, which are the topic of this review. Although these methods are considered accurate and rigorous solutions to Maxwell’s equations, it is suggested that exploration of solutions through multiple modeling methods is most robust [1].

Some optical modeling methods are not covered here; these methods include method of moments (MoM; for background see Chapter 15 in reference [2]) and finite integral technique (FIT). These methods, while popular for other optical modeling applications, have not been used widely to model anti-reflective subwavelength structures, and will therefore not be discussed.

### 1.2. Background on Anti-Reflective Sub-Wavelength Structures

**Figure 1:**
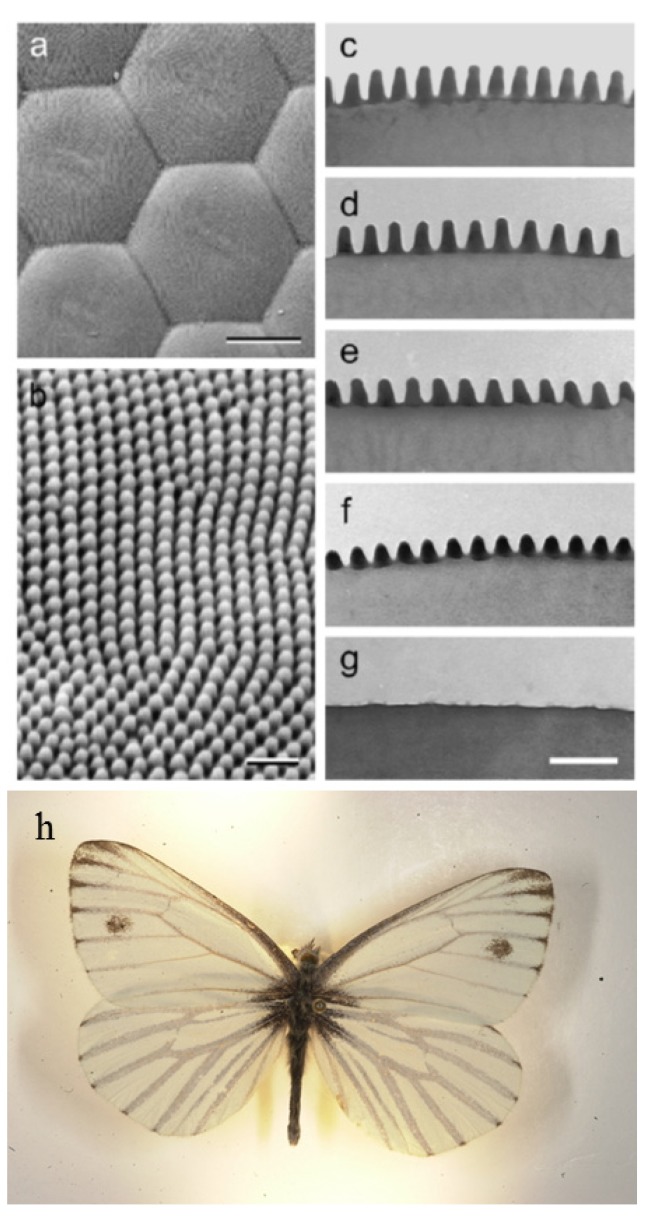
Example images of moth-eye structures found in nature. The scale bars are (**a**) 10 µm; (**b**–**g**) 500 nm. Example image of *Pieris napi* (**h**). Figure 1a–g reprinted with permission from reference [4], Copyright 2006 Elsevier; Figure 1h reprinted with permission from reference [5], Copyright 2004–2013 John Pickering.

Anti-reflective subwavelength structures are a type of biomimicry. It was found that the eyes of moths and butterflies include a surface layer of regular nipple arrays that reduced light reflection from the air/eye interface. Stavenga *et al*. [3] characterized the ARSWS on the eyes of 19 diurnal butterfly species and found that the conical nipple arrays had periods of 180 to 240 nm with heights up to 230 nm, many of which are appropriate anti-reflective structures (see Figure 1).

ARSWSs can be tapered structures with a gradient index of refraction (GRIN), non-tapered structures, sparse or densely packed, and/or made of the same material as one of the interface materials or a different material entirely. These characteristics are chosen as a balance between an ideal ARC for the situation at hand and the manufacturability of that ARC.

The least complicated ARCs are quarter wavelength, intermediate index thin films [6]. These ARCs target specific wavelengths by creating destructive interference between the reflections at the interfaces on either side of the film. Thin film ARCs for solar applications are ideally chosen to have thicknesses of about 125 nm, to target the peak of the air mass 1.5 (AM1.5) solar spectrum of 500 nm. For a quarter wavelength ARC to function, the index of refraction of the film must be designed according to the following equation:

*n* = (*n*_s_ × *n*_0_)^0.5^ (1)

where *n* is the index of refraction of the thin film and *n*_s_ and *n*_0_ are the indices of the substrate and atmosphere (or the neighboring materials), respectively. Thin film ARCs can come in the form of single layer anti-reflective (SLAR), double layer anti-reflective (DLAR), or multiple layer anti-reflective structures.

**Figure 2:**
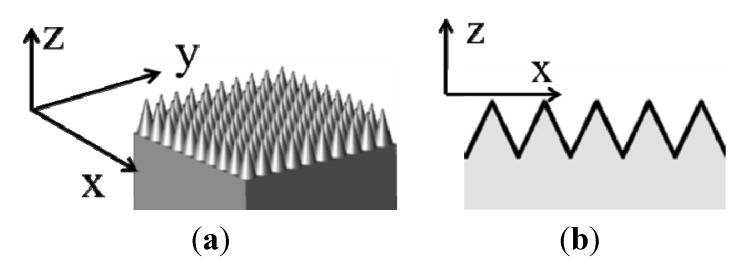
Orientation diagram of 2-D grating (3-D model, **a**) and 1-D grating (2-D model, **b**).

This review will use many terms common in the field to describe ARSWSs and optical theory. In the following pages, we will consistently refer to the *x*, *y*, and *z* directions as shown in Figure 2. A plane wave at normal incidence will be considered to be coming from the +*z* direction. Also, Figure 2 shows both a 2-D and a 1-D grating, which are modeled in 3-D and 2-D space, respectively. The angle of incidence (AOI) of the plane wave will be described as the angle from the +*z*-direction. Both of the structures shown in Figure 2 are considered gradient index (GRIN) structures, due to their continuously changing effective indexes of refraction as seen from the +*z* toward the −*z* direction.

ARSWS materials are chosen to be dielectrics to reduce reflections and absorption. Material parameters of concern in this review are index of refraction (RI) and permittivity (ε). The index of refraction is the square root of permittivity for materials with a relative permeability of one, and both RI and permittivity can be complex numbers. The real part of the permittivity describes how light slows down in a medium, which is described by Snell’s law, or the relationship between angles of incidence and angles of refraction. The imaginary part of permittivity describes the extinction coefficient and is related to light absorption. Both the real and imaginary parts of permittivity are found to be wavelength dependent.

**Figure 3:**
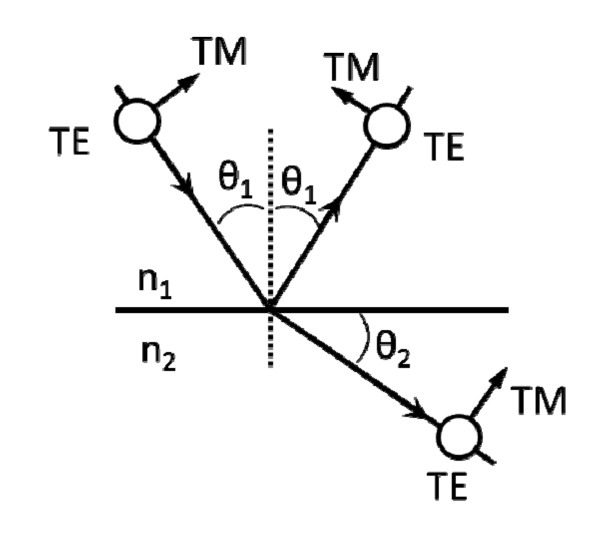
Diagram of transverse electric (TE) and transverse magnetic (TM) incident light at a non-zero angle of incidence on an interface plane for angle of incidence (AOI) < Brewster’s angle.

The optical models covered in this review use plane waves as incident light, either polarized or unpolarized. Unpolarized plane waves are equivalent to the averaging of the two polarizations of the plane waves, transverse electric (TE) and transverse magnetic (TM). As shown in Figure 3, TE light (transverse electric) has its E-field aligned with the plane of incidence (or the continuous direction of a 1-D grating) and TM light (transverse magnetic) has an E-field orthogonal to that of TE. The wavevector, *k*, indicates the direction of light travel.

### 1.3. Ideal Anti-Reflective Sub-Wavelength Structures and Gradient Indexes

The most promising broadband ARSWSs are gradient-index (GRIN) nanostructures. These materials provide a smooth gradient of index of refraction at the interface between two layers. For solar modules, those layers are commonly air:glass (*n* = 1:*n* = 1.5), EVA:silicon nitride (*n* = 1.5:*n* = 2), among others. Given the few number of materials with indexes of refraction between that of glass (around 1.5) and that of air (1.0), researchers have come to rely on the effects of mixing materials at subwavelength sizes to produce intermediate indexes of refraction. When the periods of the nanomaterials are significantly smaller than the wavelength of light the effective index of refraction can be calculated using empirical or semi-analytical formulas. These formulas are covered in a later section on effective medium theory. This theory dictates that the index of refraction for a nanostructure that is smoothly tapered in the *z*-direction results in a smooth transition of effective index of refraction between the two materials of the interface, resulting in decreased reflections.

### 1.4. Properties of ARSWS Models

Inputs to ARSWS reflectivity simulations include the size, shape, period, and/or location of subwavelength structures, materials properties (real or complex permittivity and permeability) for both bulk materials and the SWS features. The simulation must include an input of appropriate EM radiation, usually a polarized plane wave, and a way to detect the power of reflected and transmitted EM energy. The simulation must have appropriate boundary conditions to describe the material being simulated, either periodic or absorbing conditions on *x* and *y* and absorbing on *z*. The absorbing conditions used in several techniques, including FDTD and FEM is a perfectly matched layer, a mathematical method of attenuating any signal that is traveling normal to the boundary, which deletes artificial reflections in the computational domain.

**Figure 4:**
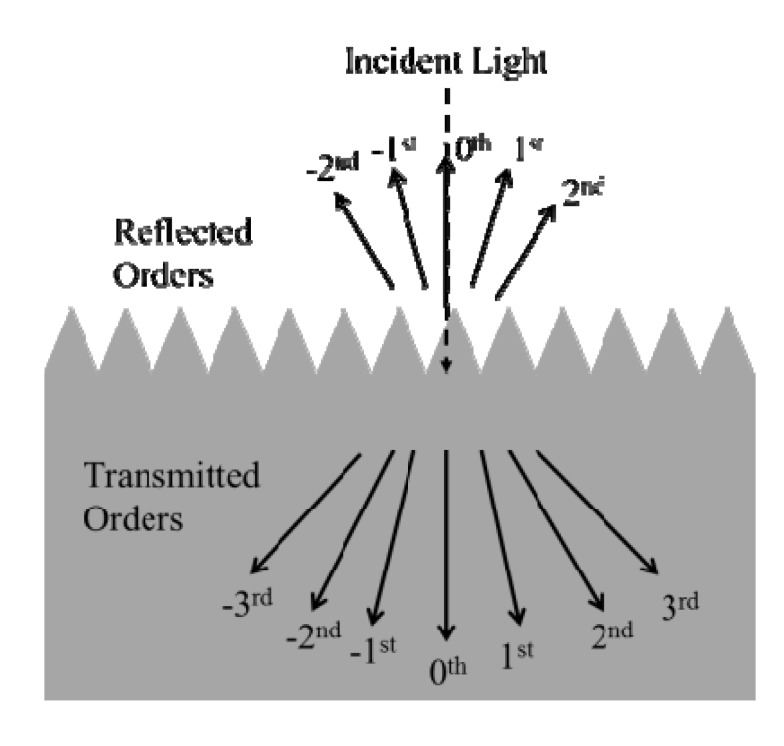
Diffraction orders on a 1-D grating at normal incidence.

Although many optical modeling methods have been developed for diffraction gratings to handle higher order reflections and refractions, sufficiently small ARSWSs should primarily require consideration of zeroth order reflections and transmissions [7]. For frequency-based numerical modeling methods this characteristic simplifies the required calculations, though exploring simulations of higher order diffraction calculations can confirm the accuracy of the method. Figure 4 displays a 2-D simulation of normal incidence on a 1-D grating and the orientations of the zeroth and higher order diffractions.

ARSWS reflectivity simulations can be performed over a range of wavelengths, angles of incidence (AOI), and polarization (TE or TM). For solar module applications ARSWS are often modeled between 400 and 800 nm or 400 and 1200 nm, with 400–800 nm being the peak solar input at AM1.5 and 400–1200 nm being the useful range of encapsulated solar modules based on the extinction coefficient of the lamination materials below 400 nm and the bandgap of silicon solar cells above 1200 nm.

For stationary (non-tracking) solar modules the behavior of an ARC at a variety of angles of incidence is important. Chuang *et al*. [8] found that moth eye structures exhibit inverse polarization at the Brewster angle. Although these structures eliminated the Brewster effect, they also have potential to decrease reflectance at higher angles of incidence, which can be important during morning and evening solar electricity collection. All methods covered in this paper are capable of simulating non-zero angles of incidence.

### 1.5. Commercial EM Modeling Software Packages

There are commercial software packages available for many electromagnetic modeling techniques. Though some were originally intended to model EM situations such as antennas or EM interference in electric circuits, most can be used to model reflection and transmission of anti-reflective coatings as well. A non-exhaustive list of software for FDTD simulations is XF by Remcom, FDTD Solutions by Lumerical, Meep from MIT, OptiFDTD, EM Explorer, and FullWave by RSoft Design Group. CST Studio uses the Finite Integral Technique (FIT), Transmission Line Matrix method (TLM), and Finite Element Method (FEM) for frequency and time domain solvers. High Frequency Structure Simulator (HFSS) is another commercially available FEM software, as is COMSOL. Several software packages use the Rigorous Coupled Wave Analysis (RCWA), including RODIS, Unigit, GD-Calc, and DiffractMOD by RSoft Design Group.

### 1.6. Utility of Modeling ARSWS

Several authors have described the exact size and shape of a theoretically optimal broadband anti-reflective coating [6]. However, modeling anti-reflective sub-wavelength structures is still an important field due to the difficulty and impracticality of fabricating the ideal structures. Modeling can be used to direct the fabrication of nanostructures toward a more optimal ARC, given the starting point of a structure that is more cheaply or easily fabricated.

## 2. Overview of Optical Modeling Methods

There are many different mathematical models for treating the behavior of electromagnetic radiation through subwavelength anti-reflective structures. These methods include a numerical time-based approach called the finite-difference time-domain (FDTD) method, numerical frequency-based methods of the transfer matrix method (TMM, models thin films only), rigorous coupled wave analysis (also called Fourier modal method RCWA/FMM), coordinate transfer method (C-method), and finite element method (FEM), as well as exact approaches such as Knop’s or Sheng’s handling of 2D square grooves [9,10] and geometric optics (ray tracing, which is not appropriate for sub-wavelength structures). Effective media theory (EMT) is used in conjunction with TMM to assign an effective index of refraction to discretized layers of an interface that make up subwavelength structures or other gradient index (GRIN) materials. Each of these methods has its benefits and limitations in modeling the behavior of light through ARCs that will be discussed in the following sections. Only numerical methods will be discussed in this review.

## 3. Effective Medium Theory

**Figure 5:**
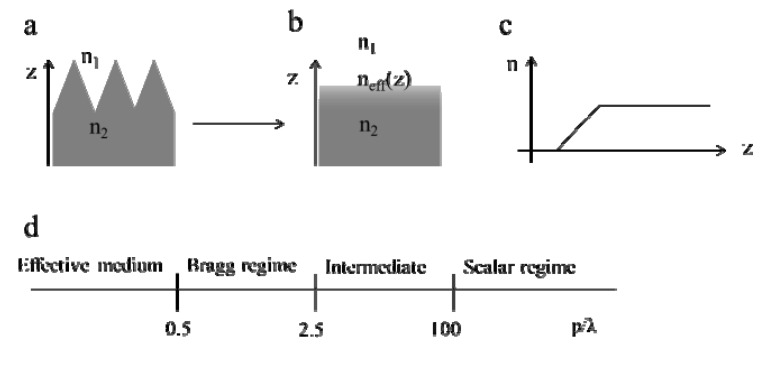
Schematic of a graded index subwavelength structure (**a**); the effective index of refraction according to how the light would interact with the material (**b**); and a graph of an approximate effective index (**c**); The various regimes of optical behavior with grating sizes are shown in (**d**), indicating that the effective medium theories are applicable for gratings whose periods (p) are much smaller than the wavelength of light (λ).

Unlike the other modeling methods reviewed in this paper, effective medium theory (EMT) is not a method for directly determining reflectance or transmittance of an ARSWS. Instead, this is a method that determines the effective index of refraction of a sub-wavelength structured geometry based on the volume fill factors of the multiple materials (see Figure 5). As shown here, EMT is only valid when the period of the texture is much smaller than the wavelength of light; some authors consider EMT only valid when the period is less than one tenth the wavelength [11]. At larger feature sizes EM waves behave in the Bragg regime, where only one or two diffraction orders are present in the diffracted (reflected or transmitted) light. Larger feature sizes require modeling of higher order diffractions and cannot considered gradient index (GRIN) materials or effective media. Other methods, such as the transfer matrix method (TMM), rigorous coupled wave analysis (RCWA) [12], or finite element method (FEM) [12] can be used to determine the reflectivity of the structured interface once the effective index of refraction is obtained for all relevant portions of a sub-wavelength feature.

EMT is used to predict the effective refractive index, and, in some cases unrelated to the subject at hand, the effective conductivity of a material. In 1956 Rytov derived an EMT solution for one dimensional periodic lamellar structures composed of two materials [13]. EMT is not used to predict the effective absorption coefficients of a material, so is most useful for dielectric materials. EMT also does not account for the size, shape, or arrangement of subwavelength textured materials. The polarization of the incident wave on a grating is generally not accounted for, except by Brundrett *et al*. [14,15] for the case of high special frequency dielectric gratings and by Lalanne and Lemercier-Lalanne for one-dimensional gratings.

**Figure 6:**
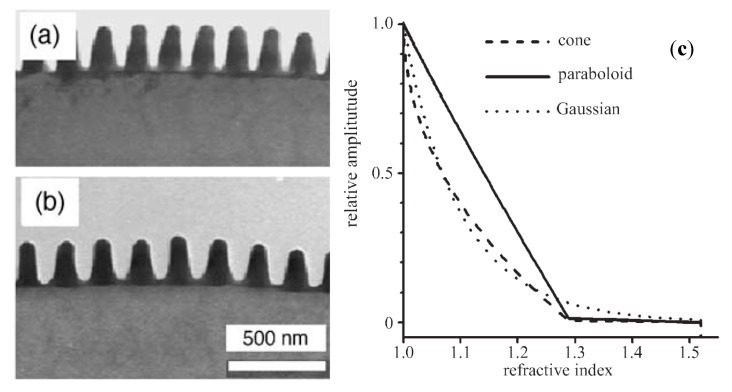
TEM images of moth eye nipple arrays (**a**,**b**) and (**c**) the effective index of refraction for three nipple types that exhibit graded index (gradient index of refraction, GRIN) behavior. Reprinted with permission from reference [6], Copyright 2010 Elsevier; and reference [4], Copyright 2006 The Royal Society.

The effective index of refraction of a subwavelength mixture of materials falls between the upper and lower indices set by the bulk values for the constituent materials [see Equations (2) and (3) as well as Figure 6] [12].

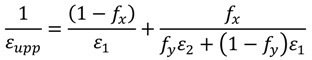
(2)

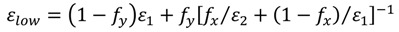
(3)
where ε_upp_ and ε_low_ are the highest and lowest possible permittivities of the mixture and ε_eff_ lies somewhere between these two, ε_1_ and ε_2_ are the permittivities of the surrounding material and substrate, and *f_x_* and *f_y_* are the fill factors of material 2 in the *x* and *y* directions. These guidelines can be used to determine the effective index of refraction of gratings.

### Effective Medium Approximations

The most common effective medium approximations (EMAs) are the Bruggeman’s model, the Maxwell-Garnett Equation, and the Lorentz-Lorentz model (See Table 1). These three methods assume approximately spherical subwavelength grains with fill factors *f* and (1 − *f*). The Maxwell-Garnett method is used for homogeneous mixtures of two materials, where the material with the refractive index (RI) of RI = *n*_2_ is surrounded by that with RI = *n*_1_. This method was originally developed to describe the behavior of light travelling through glass with a small volume fraction of silver, copper, or gold nanoparticles [16]. Material described by this method is assumed to have low volume fractions, where material 2 is in lower quantity and is surrounded by material 1. If these assumptions are met, then the effective refractive index can be calculated using the Maxwell-Garnett equation found in Table 1. For materials with more than two components the Bruggeman or Lorentz-Lorentz models are appropriate estimations of the effective index (see Table 1). In these models the effective media are assumed to consist of two or more materials, for which the effective index of refraction between those of the constituent materials. More information on these three effective medium approximations and their microscopic- or macroscopic-based derivations is described by Aspnes [17].

**Table 1:** Common effective medium approximations (EMA) methods.

Method	Model	Notes
Maxwell-Garnett [16,18]	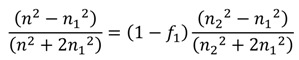	Original model for effective index of refraction (RI), assumes homogenous mixture of low volume fraction of spherical sub-wavelength structures (SWS) for material 2
Bruggeman [19]	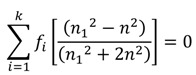	Describes effective RI for any number, *k*, of constituents in a homogeneous mixture
Lorentz-Lorentz [17]	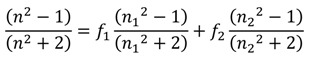	Can be extended to more than two constituents by adding more terms

Lalanne and Lemercier-Lalanne derived rigorous semi-analytical effective medium approximations for normal incidence waves on one- and two-dimensional periodic structures [15]. The authors used a Fourier expansion method on the permittivity of the gratings to derive an EMA for TE polarization of symmetric or asymmetric 1-D structures, a closed-form second-order EMA for TM polarization, and closed-form zeroth- and second-order EMA for 2-D symmetric periodic structures (see Table S1). The results for effective index inserted into the transfer matrix method are compared to those determined by rigorous coupled wave analysis with very good agreement.

**Figure 7:**
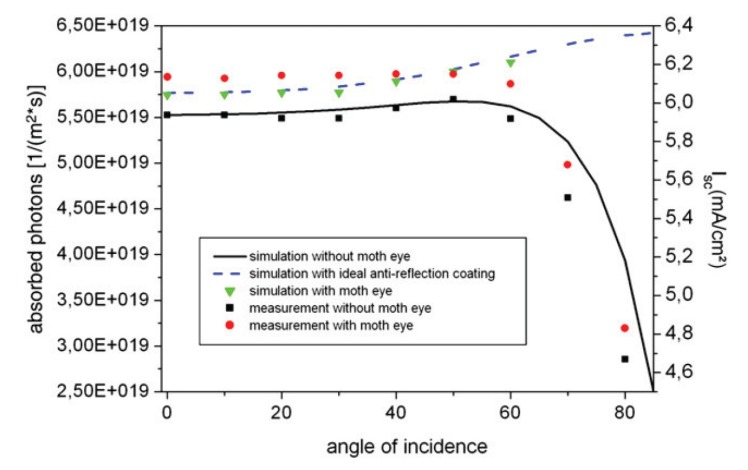
Simulation with effective medium theory (EMT) and measurement results of short circuit current from organic solar cells with no ARC, with a moth eye ARC, and with an idea GRIN structure ARC. Reprinted with permission from reference [20], Copyright 2008 Elsevier.

Forberich *et al*. [20] used effective medium approximations to model moth eye and theoretically ideal anti-reflective subwavelength structures (see Figure 7). The authors used calculated theoretical reflectivity results convoluted with external quantum efficiency measurements under a solar spectrum to determine the theoretical current output for their organic solar cells with moth eye or ideal anti-reflective coatings. They found that the EMT simulations of moth eye coatings (green triangles in Figure 7) were comparable to the measured moth eye coated currents (red dots) up to a 60° angle of incidence. Simulations and measurements without ARCs on these organic solar cells were in very good agreement (black line and dots). Forberich *et al*. [20] explain the small discrepancies between measurement and simulation as due to the imperfect matching of the moth eye material and the solar cell substrate; if the moth eye structures were made of the same material as the solar cells then the AR properties would improve.

**Figure 8:**
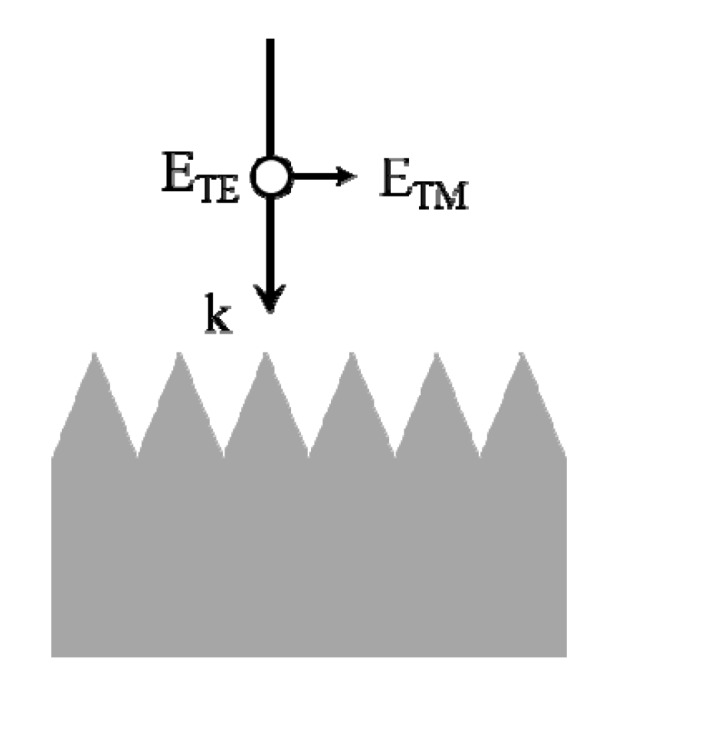
TE and TM polarization hitting a 1-D grating. The wavevector is indicated as *k*.

Brunner *et al*. [15,21,22] reviewed the derivation of polarization dependence of effective medium approximations on 1-D gratings. For 1-D gratings TE polarized EM radiation has its E-field lined up with the non-spatially dependent axis in the plane of the grating (see Figure 8). The effective index of refraction can be expanded in a power series. The two polarizations can be described by power series with up to the second order retained:

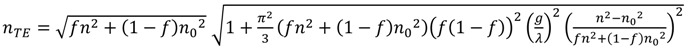
(4)

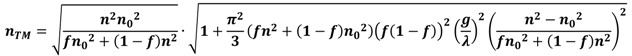
(5)
where *g*/λ is the period-to-wavelength ratio and *f* is the fill factor.

There are several commonly reported simple mathematical gradient index profiles found in the literature. Of these, the quintic and exponential sine profiles [Equations (6) and (7)] are considered to have the most ideal broadband anti-reflective properties [6].

Quintic index profile:

*n* = *n*_0_ + (*n_s_* - *n*_0_)(10*t*^3^ - 15*t*^4^ + 6*t*^5^) (6)

where *t* is the distance from the substrate in the GRIN region.

Exponential sine profile:

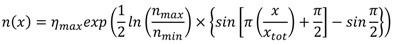
(7)
where *x* = ∫^*z*^_0_ *n(z')dz'* and *x_tot_* = ∫^*d*^_0_ *n(z')dz'* are the optical distance from the substrate and the total optical thickness. The metric thickness axis, *z*, increments from the substrate toward the top of the ARSWS and d is the total thickness of the ARSWS (GRIN region). The effective index of refraction is represented by *n*, while η is the effective admittance at an oblique angle of incidence. Structures with a quintic index profile are called “Klopfenstein structures” [21], which are shown in the rigorous coupled-wave analysis section of this review.

Other common GRIN profiles explored in the literature include the linear [Equation (8)] and cubic [Equation (9)]:

Linear index profile:

*n* = *n*_0_ + (*n_s_* - *n*_0_)*t* (8)

Cubic index profile:

*n* = *n*_0_ + (*n_s_* - *n*_0_)(3*t*^2^ - 2*t*^3^) (9)

where *t* is the distance from the substrate, *n*_0_ is the index of a vacuum, *n*_s_ is the index of the bulk ARSWS material. Another profile type, the super-Gaussian, is give by:

Super-Gaussian:

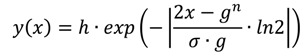
(10)
where σ is the width of the profile, the exponent *n* is a parameter that models flatness (the super-Gaussian order), height h and grating period g are scaling factors in the *x*- and *y*-directions. This describes the shape of the gradient index, rather than a direct solution for the index as in the other profiles in this section. The super-Gaussian topography profile describes the gradient index of a sinusoidal surface [21].

**Figure 9:**
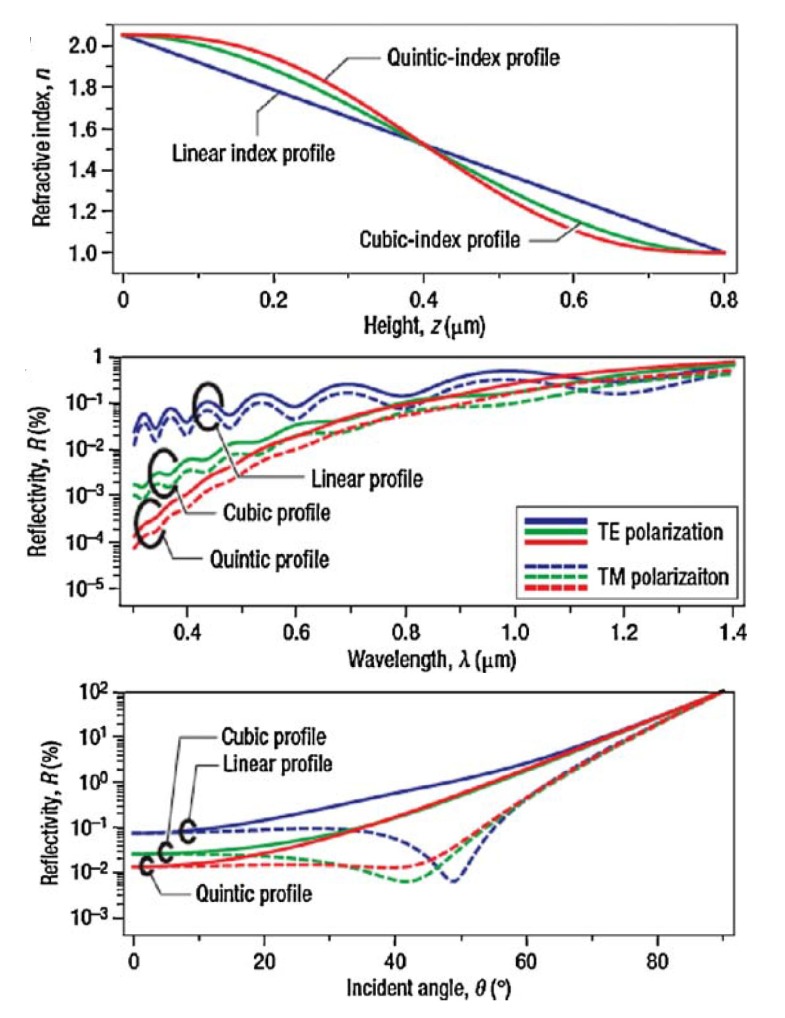
Reflectivity of several GRIN structures over a range of heights, wavelengths, and angles of incidence. Reprinted with permission from reference [23], Copyright 2007 Nature Publishing Group.

Xi *et al*. [23] described the shape of the gradient index and the effects of height, wavelength, and angle of incidence on the associated reflectance of the GRIN structures (see Figure 9). The structures included the linear, cubic, and quintic index profiles. The quintic index profile performed the best of these three shapes with an overall reflectance of less than 0.1% in the visible region. TE and TM polarizations are shown for the wavelength and angle of incidence dependence. The shapes in this experiment were approximated by 1000 homogeneous layers, which were used to calculate the reflectance by the transfer matrix method.

## 4. Time-Based Optical Modeling Methods

### Finite-Difference Time-Domain

The finite-difference time-domain (FDTD) numerical modeling method is considered to be one of the most accurate and simple rigorous methods to model anti-reflective properties of sub-wavelength structures. Though it is computationally intensive, the FDTD method handles any arbitrarily shaped structure naturally using an explicit numerical solution to Maxwell’s curl equations.

**Figure 10:**
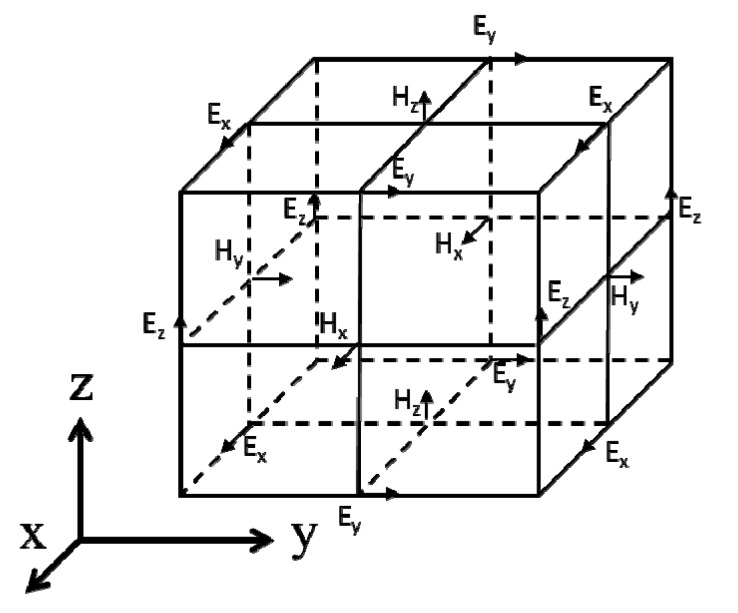
Yee cell. Arrows indicate the direction of the *E* or *H* field that is calculated at each point.

The FDTD method was first introduced in 1966 by Yee and was furthered by Taflove [24,25]. Yee developed the mathematical approach to spatially discretize the computational space into what is now known as a Yee cube (see Figure 10). The Yee cube is the unit cell of the equally offset electric and magnetic field computation points. The FDTD method, described thoroughly in Taflove’s book “Computational Electrodynamics: the finite-difference time-domain method” [26] was first developed to model electromagnetic (EM) radio waves. However, due to the simple and versatile approach, it is able to naturally handle any EM modeling situation given sufficient computing resources.

Prior to the turn of the millennium, lack of computing resources was a limiting factor to analyzing ARSWS using the FDTD method. Yamauchi *et al*. [27,28] paved the way with their 1993 and 1996 publications that modeled simple 2D thin film ARCs. In 2004, Yang *et al*. [29] first used the FDTD method to model a 3D nanoporous structure. The introduction of exponentially increasing computing resources in the 2000’s enabled the EM modeling community to utilize FDTD to its greatest advantage; researchers were finally able to model the behavior of light at an interface of any texture, size, or shape, regardless of its regularity, with only the knowledge of bulk material properties. Calculations in the time domain avoid the problem of the single wavelength restriction of other methods. Given sufficient computing power FDTD is also capable of accurately modeling structures of any size to wavelength ratio, whereas EMT requires wavelengths much longer than the interface texture and geometric optical approaches require wavelengths much shorter than interface structures. However, FDTD is highly demanding of computer processing, memory, and storage abilities compared to frequency-based or non-spatially discretized methods.

The FDTD method is derived from Faraday’s and Ampere’s laws [Equations (11) and (12)] as well as the relationships between the electric field (*E*), the electric displacement field (*D*), the magnetic field (*B*), and the auxiliary magnetic field (*H*) [Equations (13) and (14)]. These four equations are used to derive Maxwell’s curl equations [Equations (15) and (16)]:

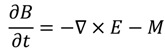
(11)

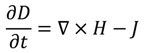
(12)

*D* = ε_*r*_ε_*0*_*E* (13)

*B* = µ_*r*_µ_*0*_*H* (14)

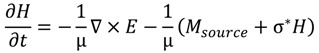
(15)

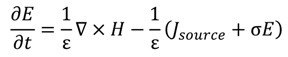
(16)
where ε is the permittivity, µ the permeability of the medium, and σ is the conductivity. When conductivity is zero the Maxwell’s equations can be rewritten as

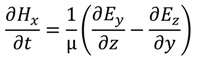
(17)

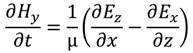
(18)

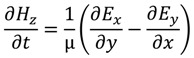
(19)

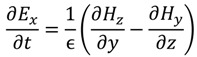
(20)

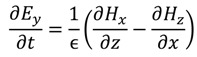
(21)

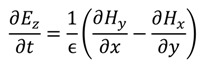
(22)

Equations (17)–(22) can be discretized using the central difference approximation to produce six algebraic equations that describe the behavior of EM waves in three dimensions. These equations are solvable for the electric and magnetic fields in each dimension: *E_x_*, *E_y_*, *E_z_*, *H_x_*, *H_y_*, and *H_z_*. To carry out the FDTD calculations, permittivity and permeability properties are assigned to each point in the computational grid and boundary and initial conditions are set (described by Taflove [26]). The discretized *E* and *H* equations are then solved alternately at each half time step, with the current E values depending on previous and adjacent *H* values and *vice versa*. Using this scheme, one can timestep through an entire simulation, resulting in electric and magnetic field values at each point for each timestep. For ARSWS analysis, these time-based data points are discrete Fourier-transformed (DFT) to the frequency scale [30]. The power of the EM waves is then calculated by squaring the absolute value of the DFT result and is multiplied by the index of refraction to account for the change in velocity in a non-vacuum medium if applicable. Reflectance and transmittance are found by normalizing the power against the input EM power.

Appropriate boundary conditions must be applied to the computational boundaries to avoid artificial reflections within the domain. Implementing boundary conditions in FDTD for ARSWS analysis is normally done one of two ways: an absorbing boundary condition or a periodic boundary condition. Periodic boundary conditions are simple and allow for modeling of an infinitely large array of ARSWS features. Absorbing boundary conditions (ABCs), which are necessary for all non-periodic boundaries, function to attenuate the EM signals at the interface. The most commonly used ABC is the perfectly matched layer (PML), which was introduced by Berenger in 1994 [31]. The PML functions to anisotropically attenuate all EM intensity that is traveling in the direction toward the boundary, effectively eliminating any artificial reflections from that surface.

**Figure 11:**
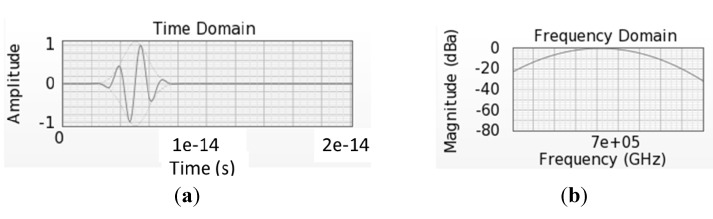
Time-based *E*-fields introduced as a plane wave (**a**) to simulate a range of frequencies (**b**).

Introducing a plane wave into an FDTD simulation is done by setting the *E* and *H* values in one plane to appropriate non-zero values for a period of time, either a short pulse or a continuous source. All values in the plane must be the same at any given time point to ensure plane wave functionality. The signs of *E* and *H* must be chosen to propagate the EM wave in the desired direction following the right hand rule. Plane waves can be introduced as monochromatic waves (with the *E* and *H* fields oscillating in time at the appropriate rate) or as a distribution of wavelengths. An example of introducing a Gaussian distribution of wavelengths into one simulation can be seen in Figure 11.

In FDTD materials are defined only by their permittivity and permeability properties. Most ARSWS FDTD studies are based on dielectric materials, thus, the relative permeability is one and absorptive losses can be ignored. This is especially true when analyzing dielectric materials that are optically thin. Most studies also ignore dispersion, or the effect of wavelength on the permittivity, as permittivity of the commonly studied materials is relatively constant over the AM1.5 range. FDTD does not handle dispersion naturally, as multiple wavelengths are input simultaneously, but modern computing programs have been improved to include dispersion.

Programming FDTD simulations manually is feasible and is fully supported by Taflove’s text on the subject [26]. However, there are several commercially-available or free software products that make FDTD simulations significantly easier to use, including XF by Remcom, FDTD Solutions by Lumerical, MEEP (open source), OptiFDTD, EM Explorer, FullWave by RSoft Design Group, and Electromagnetic Template Library. Commercial software often includes a user-friendly GUI and CAD modeling tools for drawing 2D or 3D materials.

Whether using in-house or commercial software, several guidelines must be taken into account when setting up an FDTD simulation. To be accurate, the software requires at least ten computational cells per wavelength. The time step is usually chosen so that there are at least 20 timepoints per wavelength. There must also be at least three calculation points across any feature that one is expecting to model; failure to comply with this requirement often results in the improper modeling of the tips of pointy nanostructures [32]. This pixilation effect would produce an artificially abrupt change in the effective refractive index at the pointiest parts of the ARSWS. Deinega *et al*. [33] reported a subgrid smoothing method to account for this effect, which improved the modeling of fine features. Even with this drawback, several authors reported the choice of FDTD over the rigorous coupled wave analysis (RCWA) method due to RCWA being oversimplified for some 3D models [30,34,35].

**Figure 12:**
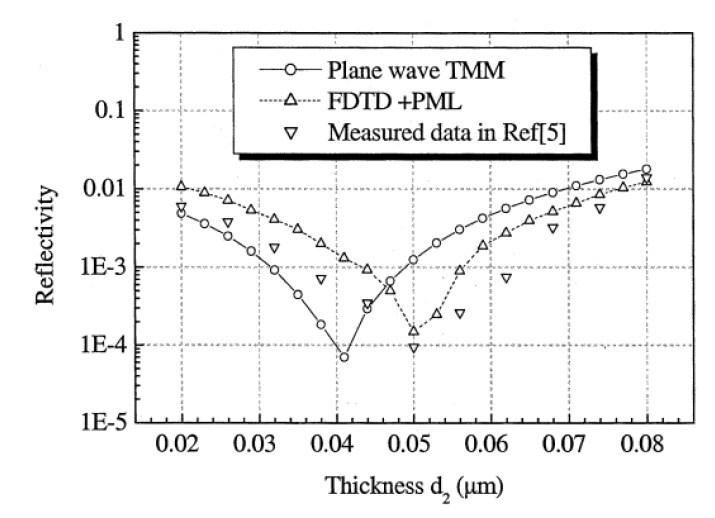
Reflectivity calculated from the finite-difference time-domain (FDTD) and transfer matrix method (TMM) methods compared to experimental data shows that the FDTD method is more accurate than the TMM method. Reprinted with permission from reference [36], Copyright 2003 IEEE.

Several groups have used the FDTD method as an accurate method to fine-tune the designs of ideal anti-reflective interfaces. FDTD has been used in conjunction with the transfer matrix method (TMM); Feng *et al*. [36] developed a space mapping technique that applied both TMM and FDTD to converge on an optimal design for the thicknesses of a multiple thin film layered ARC. Feng *et al*. [36] compared the calculated reflectivity from FDTD, TMM, and experimental data to show that the FDTD method was more accurate than the TMM method for their multiple thin film simulations (see Figure 12). Li *et al*. [37] and Zhou *et al*. [38] both also used TMM with FDTD to design antireflective and waveguide structures. The transfer matrix method is very efficient, but has limited accuracy due to its inherent approximations, while the FDTD method is versatile and accurate, but time consuming [36].

**Figure 13:**
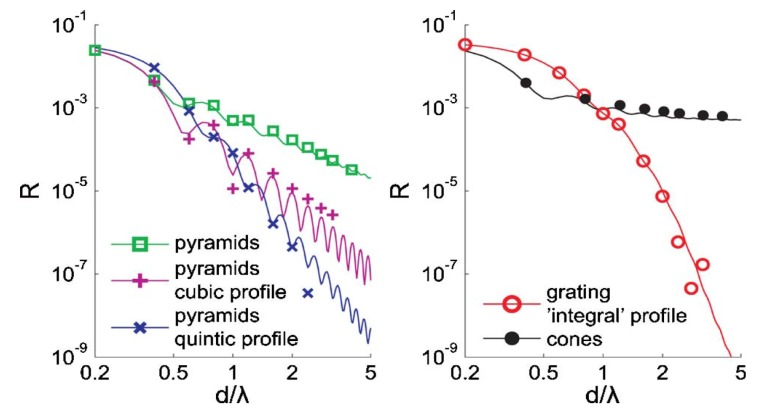
Reflectivity results from graded-index films with an integral RI profile (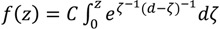), square pyramids with linear and quantic RI profiles closely packed in a square lattice, and cones closely packed in a triangular lattice from EMT (lines) and FDTD (points) calculations. Reprinted with permission from reference [32], Copyright 2011 Optical Society of America.

**Figure 14:**
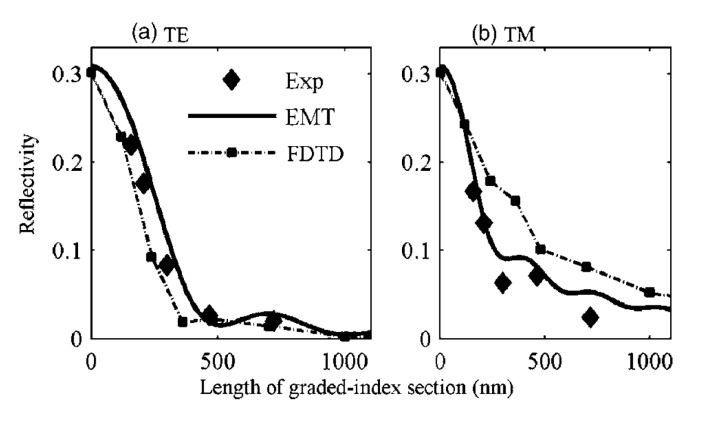
Comparisons between reflectivity calculated by EMT, FDTD, and experimental results for GRIN structures. Reprinted with permission from reference [40], Copyright 2007 Optical Society of America.

Several groups used both FDTD and TMM, but refer to the TMM simulations as “effective medium theory” [29,32,39,40]. Effective medium theory takes subwavelength-textured structures and breaks them down into planes of effectively homogeneous “thin films”. For example, Schmid *et al*. [40] sliced their nanotexture every 1 nm for their EMT models. The traditional thin film reflectivity equation (transfer matrix method) is used to calculate the reflectivity of the overall interface. This method works well for structures much smaller than the wavelength of EM radiation, but breaks down quickly as structures approach the wavelength size and if there are any non-zeroth order reflections or transmissions (due to a diffraction grating effect). Figure 13 shows the comparison of EMT results with FDTD results (including a subpixel smoothing method) for graded-index films with an integral RI profile (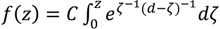), square pyramids with linear and quantic RI profiles closely packed in a square lattice, and cones closely packed in a triangular lattice reported by Deinega *et al*. [32]. FDTD results are in good agreement with EMT over the wavelengths studied except for very short wavelengths where the assumptions of EMT break down. Separate results comparing FDTD, EMT, and experimental values for reflectivity *versus* GRIN height are shown in Figure 14.

While effective medium theory requires features to be much smaller than the wavelengths of interest, geometrical optics or ray tracing is often used when feature sizes are much larger than the wavelengths [32,39,41]. Though specifically not useful for subwavelength structures, this method is often used to design anti-reflective gratings using light trapping. This method uses simple geometrical optics based on the index of refraction to calculate the behavior of light, assuming an abrupt change in index of refraction at the textured interface. Deinega *et al*. [32] report ray tracing models the short wavelength/large feature size extremes of anti-reflective designs and FDTD is still shown to have comparable, accurate results. However, due to the requirements of having many computational points per wavelength in space for this method it may not be ideal to model particularly large structures with FDTD due to processing power and data storage constraints.

Chen *et al*. [7,8] produced two studies that analyzed cones and pyramids using both RCWA (discussed in a later section) and FDTD. Ichikawa used both FDTD and the Fourier modal method (FMM/RCWA) to design two dimensional regular and random triangular gratings as early as 2002 [42]. The Fourier modal method was used to simulate the triangular gratings as a stack of twenty slabs and was used to verify the FDTD results. The author found that, while randomizing the triangular gratings in shape-, space-, or depth- modulated structures did not increase the AR properties of the SWS, the randomization did relax some of the subwavelength requirement for the ARC, which relaxes some of the fabrication constraints. They also determined that FDTD predicts higher reflectance than does zeroth order FMM, likely due to the neglect of higher order reflections in the FMM model. RCWA is a rigorous simulation method, but some authors have felt that it is too oversimplified to accurately model some 3D structures [30,34,35], especially those that are aperiodic.

As technology improvements have made 3D FDTD modeling faster and easier, more authors are performing several FDTD simulations to sweep across a range of feature properties in an attempt to design an optimal AR structure (Table S2). Over primarily the last decade researchers have used FDTD to model thin films [27,36], nanoporous materials [29], regular and random 2D triangular gratings [42], cones [8,32,33,35,39,43,44], pyramids with a variety of base shapes [30], semi-spheres [30,34], rounded cones [43,45], nanoholes [46], and nanorods/nanowires [47,48]. As is reviewed in Chattopadhyay *et al*. [6], gradient index materials, or tapered nanostructures, generally perform the best as broadband anti-reflective interfaces. Thus, most of the FDTD simulation sweeps in the last decade have focused on sizes and shapes of pyramid, cone, or other nipple-like arrays of nanostructures. The ARSWS with the best broadband AR properties for wavelengths between 400 and 800 nm were found to be closely packed tapered nanostructures, cones or pyramids, with periods around 300 nm and lengths between 300 and 600 nm at about 0.3% reflectivity [34].

**Figure 15:**
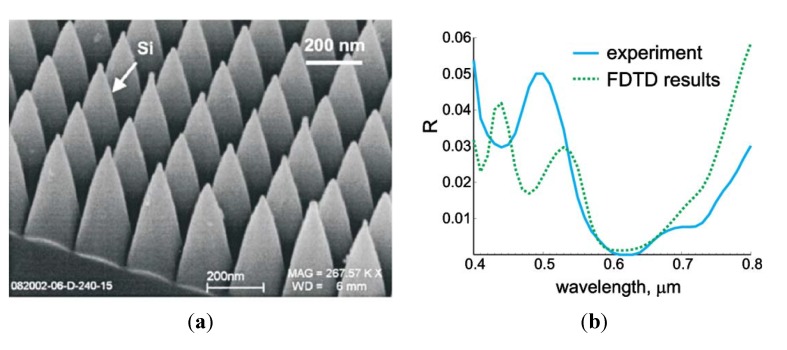
SEM image of square packed silicon cones (**a**) and (**b**) comparison of FDTD (dotted) and experimental results (solid). Reprinted with permission from reference [32], Copyright 2009 Optical Society of America.

The FDTD method has been verified by experimental results by several authors (Table S3). Deinega *et al*. [32] found the results of the FDTD simulations to be highly comparable to experimental results (Figure 15). Other authors have compared modeling and experimental results for pyramids [7], cones [45,48,49], hexaganol nanorods [47], round nanowires [48], tapered nanorods [47], and v-shaped nanoholes [46]. Although closely packed pyramids with high aspect ratios are known to have a smoother RI profile at the interface with the bulk material than cones due to their smooth fill percent profile, and hence theoretically lower reflection given the correct RI profile, they can be difficult to fabricate. Chen *et al*. [7] were able to fabricate what was effectively an array of hexagonal pyramids using a polystyrene nanosphere colloidal lithography technique. Their simulations predicted that the nanotexture would reduce the reflectance of the silicon to less than 1% and they were able to synthesize pyramidal structures with a reflectance of less than 1.5%.

**Figure 16:**
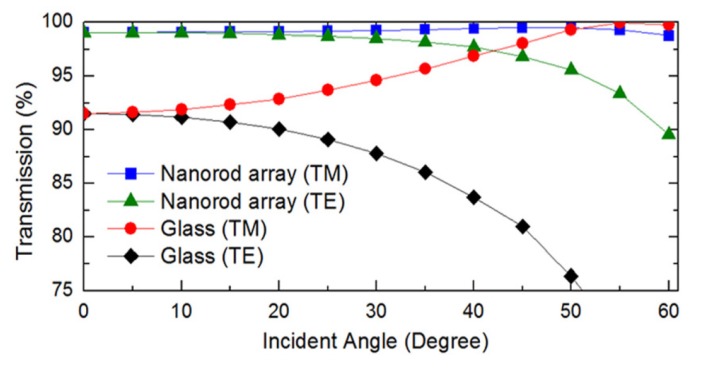
Angle of incidence FDTD simulations for nanorod arrays in TE and TM polarization [47]. Reprinted with permission from reference [47], Copyright 2011 AIP Publishing.

FDTD has been used in literature to obtain reflectivity information about ARSWSs at non-zero angles of incidence. Deniz *et al*. [47] studied the effects of angle of incidence and plane wave polarization. They modeled hydrogen silsesquioxane nanorods that exhibited less than 2% average reflection over 400 to 800 nm wavelengths for between 0 and 60 degrees of incidence from normal in TM polarization (see Figure 16). Some authors used FDTD to model embedded nanoparticles in silicon to enhance light scattering. Mokkapati *et al*. [50,51] modeled EM fields around metallic nanoparticles to increase light scattering for light adsorption in solar cells, though they reported E-field intensity, not reflectivity. Nagel and Scarpulla [52] modeled the light-trapping effects of embedding silica nanospheres in thin film silicon solar cells. Two groups also used the FDTD method to model the laser ablation process to produce AR nanomaterials [53,54].

The FDTD method has been shown repeatedly to be a versatile, simple, and accurate modeling method for 2-D and 3-D modeling of anti-reflective subwavelength structures. This method can be accurate over any wavelength and feature size combination and can be used with any structure, regular or irregular. The FDTD method does not naturally handle dispersion, though refractive indexes in the visible wavelengths are relatively constant, and thus the modeling results from FDTD have been shown to match experimental results satisfactorily well. This can be overcome by inputting one wavelength per simulation and assigning the appropriate wavelength-dependent optical properties to the material. Computational resources have, in the past, limited the utility of FDTD modeling, though with the introduction of newer computing technologies these limitations are becoming fewer.

FDTD has been found to have convergence problems when attempting to calculate dispersion, with some metal components [55], or when modeling some features whose sizes approach the wavelength of EM radiation. These disadvantages have primarily been overcome by implementation of new algorithms in commercial software to reduce divergence and with faster hardware to handle longer simulation times. Also, for analysis of reflectance of ARSWS, FDTD requires post processing of time-based *E*-field data to obtain values for reflectance. This can either be a part of the modeling software or separate post-processing software.

## 5. Frequency-Based Optical Modeling Methods

### 5.1. Transfer Matrix Method

The transfer matrix method (TMM) is a simple approach to modeling waves passing through layered media. Appropriate only for thin film ARC modeling, this method employs continuity boundary conditions between layers of material and wave equations to describe the electric fields or reflectance and transmittance values across each layer. Continuity requires that the fields at the interface between two materials be the same in each material. Then, if the electric field is known at the beginning of the layer a transfer matrix based on the wave equation can be used to determine the electric field at the other end of the layer (see Section 6.2.1 in reference [56]). Both reflected and transmitted waves are considered to calculate the overall electric fields.

**Figure 17:**
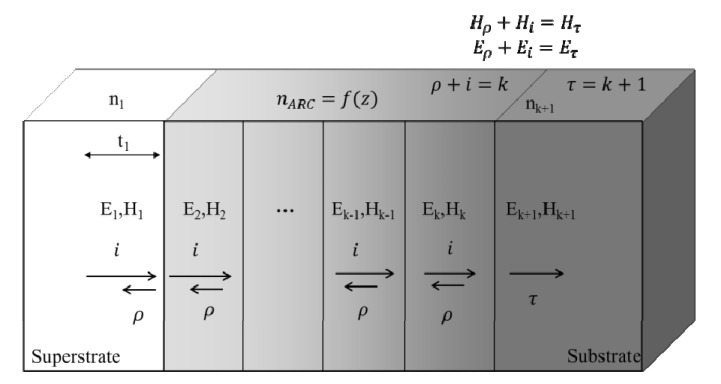
Geometry of the transfer matrix method. Each layer left to right represents a separate, homogeneous material. *H* is the magnetic field and *E* is the electric field. The thickness, *t*, of each layer is drawn. The RI is labeled as *n*, reflected wave energy as ρ, transmitted energy as τ, and *k* is a designation of layer position (this diagram includes layers 1 through *k* + 1).

Figure 17 demonstrates the theory behind the transfer matrix method. In this figure the left hand side contains the incident EM wave and the right hand side depicts the transmitted wave. The center blocks represent hypothetical layers of a thin film GRIN structure, each with its own index of refraction. In each block the forward and backward diffracting EM waves are added up to describe the total fields.

Below is a summary of the mathematical derivation for TMM found in Condon and Odishaw’s Handbook of Physics, 2nd edition [57]. It begins with Maxwell’s equations for a plane wave travelling in the *z* direction through a dielectric:

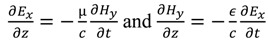
(23)
and the wave admittance, *N*, is given as the ratio of *H* to *E* (all of which have the subscripts omitted for simplicity). Given a structure layered in the *z* direction, each layer having its own index of refraction, the admittance, index of refraction, and ratio of *N*/*n* (*Q*) can be related in any layer *k* by:

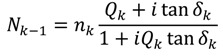
(24)
where the phase angle, δ*_k_*, is defined as *n_k_*λ_0_^−1^*t_k_*. This equation can be re-written using the definition of *N_k_* = *H_k_*/*E_k_* to give:

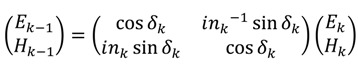
(25)

Equation (25) contains a 2 × 2 matrix called the transfer matrix; this equation calculates the input admittance *N_k_*_−1_ for any layer with index *n_k_*, thickness *t_k_*, and admittance *N_k_*. It is important to note that this method solves the system of equations in a way that appears backwards; one begins with the light transmitted through all the layers and then back calculates to obtain the input light intensity. This is required to account for the cumulative effects of light reflected at each interface.

The boundary conditions applied are:

*H_ρ_*
+ *H_i_* = *H_τ_* (26)

*E_ρ_*
+ *E_i_* = *E_τ_* (27)

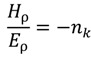
(28)
where ρ designates the reflected field, *i* is incident, and τ is transmitted. The amplitude reflection coefficient is defined as:

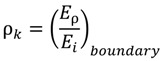
(29)

And overall yield for reflection is:

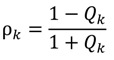
(30)

While overall transmission is:

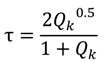
(31)
with the relative intensities of reflected and transmitted being the normal reflectance *R* = |ρ|^2^ and *T* = |τ|^2^. A transfer matrix is written for each layer of dissimilar material in the ARC.

In 1950 Abelès described a simple and fast method to determine the reflectivity from a thin film layered interface [58]. His calculations can be used to model non-zero angles of incidence. Boden and Bagnell employed the Abeles matrix method [58], a simple way to calculate spectral reflectance at any angle of incidence [59,60]. This method enabled them to optimize many layered thin film ARCs for sunrise to sunset at both the equator and in the UK. They found that optimizing for all hours of sunlight provided only modest gains in efficiency compared to optimizing for normal solar incidence at AM1.5. The differences between these optimizations were small enough that fabrication tolerances and uniformities are likely to be a more significant factor in actual performance.

The transfer matrix method has been used in conjunction with the finite-difference time-domain method to converge on an optimal multi-layered thin film ARC. Feng *et al*. [36] developed a space mapping technique that coupled the results from TMM and FDTD simulations to step through an iterative simulation to design the thicknesses and indexes of refraction for two- and four-layered ARCs. Using TMM as a rough model and FDTD as a fine model the authors were able to converge on a reflectance of less than 0.01% over a 30 nm bandwidth around 1540 nm after only three iterations of FDTD. TMM and FDTD results correlated well, though the FDTD results were determined to be more accurate.

**Figure 18:**
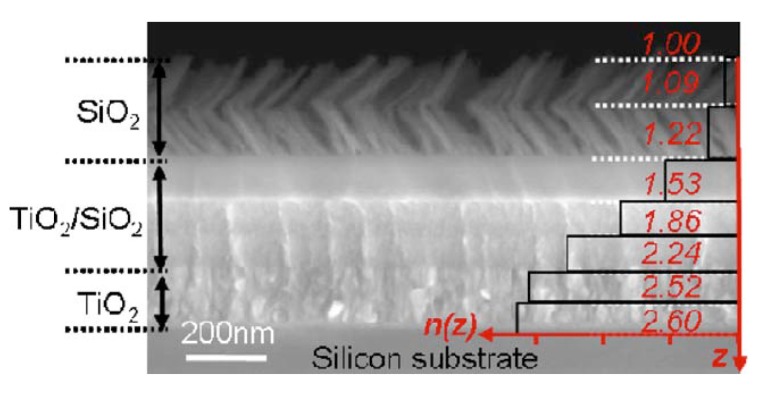
SEM image of a seven layered thin film ARC produced and modeled by Kuo *et al*. [61] using TMM. Reprinted with permission from reference [61], Copyright 2008 Optical Society of America.

Kuo *et al*. [61] used TMM to model a seven layered ARC (see Figure 18). They were able to reduce the reflectance of a silicon solar cell to 1%–6% between 400 and 1600 nm, with a measured 0°–60° angle- and wavelength-averaged reflectance of 3.79%. Forberich [20] performed optical simulations of the absorption for their solar cells using TMM, but used EMT for reflection calculations of the moth eye structures.

In the application of measuring reflectivity for thin film ARCs the transfer matrix method is a fast and simple modeling method. TMM is capable of calculating reflectance and transmittance, can handle multiple wavelengths, dispersion, and multiple angles of incidence. This method can also handle absorption via complex refractive indexes. There are several commercially available software programs that utilize the TMM method, including FreeSnell, EMPy, Luxpop.com, and Thinfilm.

### 5.2. Rigorous Coupled Wave Analysis, Fourier Modal Method, and Coordinate Transfer Method

The rigorous coupled wave analysis has been described by several authors [62,63,64]. The words of Gaylord *et al*. [64] provide a concise introduction to the method:

“The rigorous coupled-wave analysis for grating diffraction was first applied to planar (volume) gratings [65]. In these gratings, the refractive index and/or optical absorption vary periodically between the two parallel planar surfaces of the grating. In this method, the field inside the grating is expanded in terms of space-harmonic components that have variable amplitudes in the thickness direction *z* of the grating. This field expansion together with the Floquet condition (due to the periodic nature of the structure) is then substituted into the appropriate (TE or TM polarization) wave equation, and an infinite set of coupled-wave equations is formed. Using a state space representation, this infinite set of second-order equations is converted into a doubly infinite set of first-order equations. The space-harmonic amplitudes are then solved for in terms of the eigenvalues and eigenvectors of the differential equation coefficient matrix. By applying boundary conditions (continuity of the tangential components of E and H across the boundaries), a set of linear equations is formed. Truncating this set of equations so that an arbitrary level of accuracy is achieved, the amplitudes of the propagating diffracted orders and the evanescent orders may then be determined. From the amplitudes of the propagating orders, the diffraction efficiencies may be directly calculated. None of the common approximations (neglect of second derivatives, neglect of boundary effects, neglect of higher-order waves, neglect of dephasing from the Bragg angle, or small grating modulation) is used in this analysis. The method is rigorous, and any specified level of accuracy can be obtained.

Rigorous coupled-wave analysis has also been applied to surface-relief gratings [66]. In this case, the surface-relief grating is divided into a large number of thin layers parallel to the surface. Each thin layer grating is analyzed using the state variables method described above and then by applying the boundary conditions to the boundaries of each layer, it is possible to obtain the forward- and backward-diffracted wave amplitudes.”

The rigorous coupled wave analysis (RCWA) is a frequency-based, semi-analytical optical simulation method that calculates the efficiencies of transmitted and reflected diffracted orders. Working in the subwavelength domain, generally only the zeroth order of diffracted waves must be considered. This method functions similarly to the transfer matrix method, except that it incorporates lateral periodic non-uniformities of material properties in the plane of the interface [67]. RCWA is appropriate for subwavelength features or larger features, but the calculations can be simplified somewhat under subwavelength conditions.

RCWA is also known as the Fourier Modal Method (FMM) [1,68,69,70,71]. This method is also not to be confused with the rigorous modal theory, approximate two-wave modal theory, approximate multi-wave coupled-wave theory, or approximate two-wave coupled-wave theory, all of which include non-rigorous assumptions in the formulation of the models that RCWA does not contain [65]. Multiwave coupled-wave theory neglects boundary diffraction and second derivatives of the field amplitudes. Higher order waves are neglected in the two-wave modal theory (which, as stated before, is appropriate under subwavelength conditions). The assumptions of the two previous methods are all made in the two-wave coupled-wave theory.

**Figure 19:**
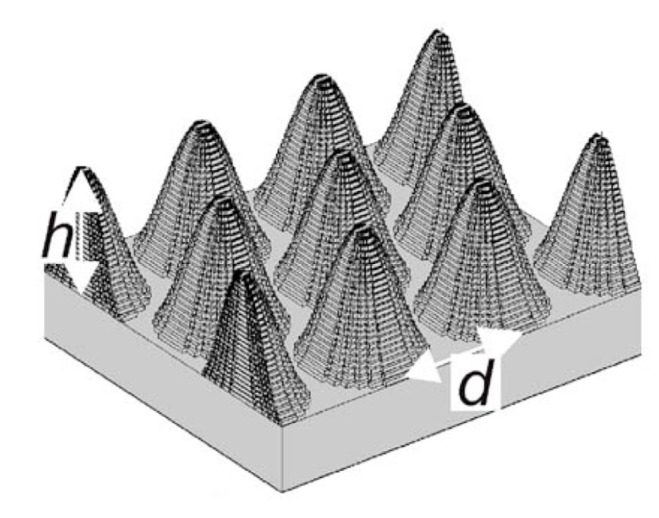
Stair step approximation of a periodic geometry as drawn in the commercially available rigorous coupled-wave analysis (RCWA) software, GD-Calc. Reprinted with permission from reference [72], Copyright 2006 AIP Publishing.

The RCWA method begins by discretizing the model geometry into a superstrate, a grating region that might include many stairstep-approximated layers to implement the grating geometry, and a substrate. An example of a stair-step-approximated geometry can be found in Figure 19. The permittivity of each layer in this geometry is described by a Fourier series. The boundary conditions of continuity between the tangential *E*- and *H*-fields at each interface between layers are then used to solve for the diffraction efficiencies for each forward and backward diffracted order. This provides the user with a simple account of transmission efficiency without the need for data post processing; the efficiencies of each forward diffracted wave are summed to obtain total transmission efficiency [60].

The RCWA method, its accuracy, and example calculations are provided by Hench and Strakos [63]. The obvious question of level of accuracy of the modeling method is addressed. Li developed and proved improved convergence performance of RCWA using a fast Fourier method [73], which began the use of FMM as a term for RCWA. Li found that discretizations that led to slow numerical computations were incorrect, producing a slower and less accurate model [73].

**Figure 20:**
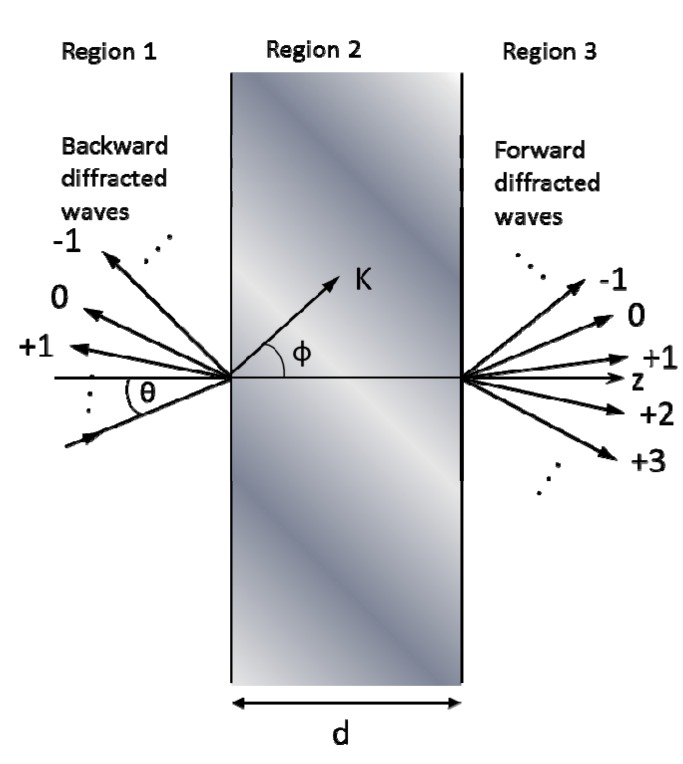
Geometry for planar-grating diffraction. Region 1 (**left**) is the region of incident light, which will contain both incident and reflected waves. Region 2 (**middle**) is the transmission grating, which will contain transmitted and reflected waves. Region 2 (**right**) is the substrate, which will contain only forward diffracted (transmitted) waves. This example uses a simple transmission grating with a sinusoidal permittivity that is oriented at an arbitrary angle from normal.

For a mathematical example of RCWA consider the early paper from Moharam and Gaylord that describes the 2D diffraction properties of a 1D grating with an arbitrarily-oriented sinusoidal permittivity function that is below a superstrate and above a substrate [65]. Figure 20 describes the geometry of their simulation. Similar to the transfer matrix method (TMM), RCWA begins with describing the electric fields in the superstrate (region 1), grating region (region 2), and substrate (region 3) as shown in Figure 20 by the summation of their reflected and transmitted waves of all orders, *i*:

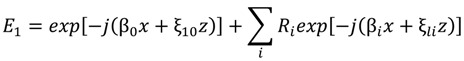
(32)
where the first exponential term represents the incident wave and the second represents the sum of all reflected diffraction orders in the superstrate region. In this equation, β*_i_* = *k*_1_sinθ − *iK*sinΦ for any integer *i* (the wave order); ξ*_li_*^2^ = *k_l_*^2^ − β*_i_*^2^ for *l* = 1, 2, or 3 (the region index); *k_l_* = 2πϵ_l_^1/2^/λ for *l* = 1, 2, or 3; λ is the free-space wavelength; *j* is the imaginary number, θ is the angle of incidence of the light, Φ is the angle of tilt of the sinusoidal permittivity of the grating, *R_i_* is the normalized amplitude of the *i*th reflected wave. The reflected wave amplitudes are some of the terms to be calculated using this method.

Inside the diffraction grating one must consider all forward and backward diffracted waves from both bounding interfaces, which can be described by:

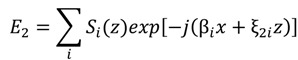
(33)
where ξ_2*i*_ = *k*_2_cosθ' − *iK*cosΦ, θ' is the angle of refraction inside the modulated region, and *S_i_*(*z*) is the normalized amplitude of the *i*th wave field at any point within the modulated region. The transmitted waves into the substrate can be described by:

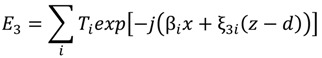
(34)
where *T_i_* is the normalized amplitude of the *i*th transmitted wave. The term coupled-wave comes from the expression of each *i*th wave (each diffraction order in each region) as a superposition of an infinite number of plane waves, which completely describes the forward and backward traveling waves of that order.

The permittivity of the grating region is described by an equation, either a Fourier expansion that describes the permittivity of one layer in a staircase approximation of a more complicated geometry, or, as in our example here, a simple equation that spatially describes the permittivity in the grating layer. Our grating follows the equation:

*ϵ(x,z)* = ϵ_2_ + Δ*ϵ*cos[*K*(*x*sinΦ + *z*cosΦ)] (35)

where ϵ_2_ is the average dielectric constant of region 2, Δϵ is the amplitude of the sinusoidal relative permittivity, Φ is the grating slant angle, and *K* = 2π/Λ, where Λ is the grating period. This equation will change to match the specifics of the grating of interest, of course, but this sinusoidal index grating is used as a simple example.

The amplitudes of the superimposed infinite sum of waves for each diffraction order are determined after considering the modulated-region wave equation (Eigen function):

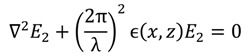
(36)

Once the permittivity and fields are appropriately described the Eigen function is solved using scattering matrices, similar to the transfer matrix used in the transfer matrix method, for the field strength of each diffracted wave.

RCWA is a highly accurate method for determining the reflectivity of a periodic grating. Numerical error comes from how well the grating can be described by a stair-step approximation, how many Fourier terms and diffraction orders are retained, and round-off errors from the numerical calculations. Due to the Fourier and Flouquet expansions used in the derivation of this method, it is necessary to truncate the expansions appropriately in order to enable fast and efficient solution with a computer. The amount of truncation chosen is determined by the accuracy required and the computing resources available [55].

**Figure 21:**
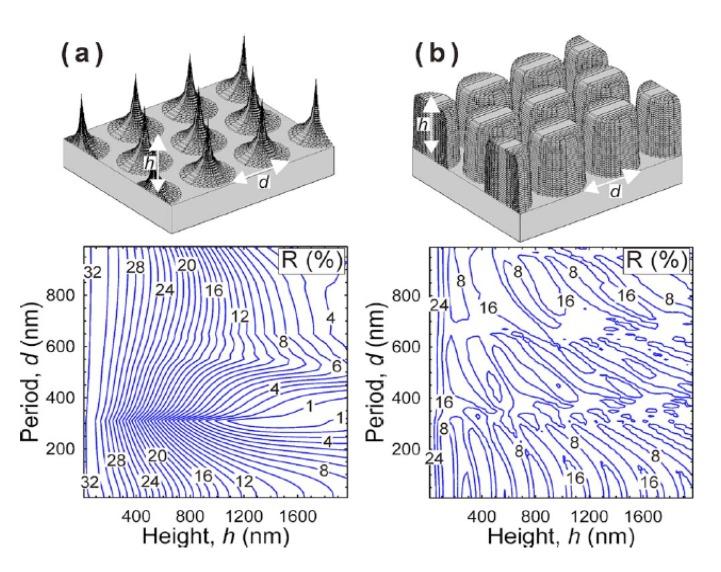
Several moth-eye-like structures with different refraction index profiles and their associated reflectance *vs*. period and height at a wavelength of 1000 nm. Reprinted with permission from reference [72], Copyright 2008 AIP Publishing.

RCWA has been used to model a large variety of periodic anti-reflective structures in three dimensions (which are considered two dimensional gratings). Most of the structures modeled with this method are hexagonally or square packed, GRIN, moth-eye-like nipple arrays [60,72,74,75,76,77,78,79,80,81,82,83] (see Figure 21) or, more specifically, tapered and truncated cones [84,85,86,87], nanocones and nanopillars [88], core/shell GRIN structures [89,90], and inverted moth eyes [67]. Many of these studies concentrated on designing the optimal period, height, or refractive index profile for the moth eye structures and some explored the effects of angles of incidence of the plane wave of light. These results show that the anti-reflection band is bounded by the period of the sub-wavelength structures and the height of the structures for any given index shape. All studies support that a smooth change in effective index of refraction from the superstrate to the substrate results in the best anti-reflective properties. Ideal index profiles are discussed in the effective medium theory section of this paper.

**Figure 22:**
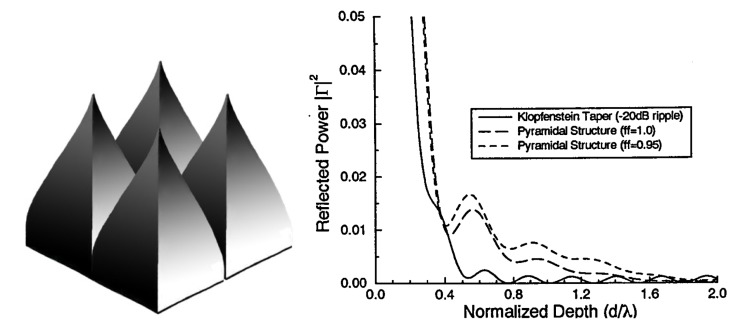
Klopfenstein structures have much better AR properties at shorter heights than do pyramids. Klopfenstein structures are shown on the (**left**), and the RCWA-calculated reflectivity *vs*. normalized depth for Klopfenstein and pyramid structures is shown on the (**right**). Right side reprinted with permission from reference [97], Copyright 1995 Optical Society of America.

Any periodic structure can be modeled using RCWA, provided the geometry can be accurately described by a stack of slices that are homogeneous in the *z*-direction for any *x*, *y* location. Many structures that are either not considered moth-eye GRIN structures or are not in a hexagonal pattern have been modeled using RCWA. Pyramids, hemispheres [91,92,93], cones, and other four-sided pyramid-like structures are modeled in a rectangular pattern using the RCWA method [11,94,95,96,97]. Grann *et al*. [97] determined that the Klopfenstein taper, shown in Figure 22, displayed similar anti-reflective properties to pyramids that were much higher, enabling the production of lower profile structures for the same AR effects. It has also been reported that for different indices of refraction the shape of the ideal Klopfenstein structure changes; higher refraction indices require more slender Klopfenstein structures [6]. Other non-moth-eye structures include honeycomb [7], cylinders or nanocolumns [8,98,99,100,101], and square holes or cubes [102]. The earliest RCWA studies focused on 1-D gratings, which can be solved in a 2-D model, including triangle [68,103,104,105,106], rectangular [14,63,64,103,107,108], slanted [66], and multilayer triangle gratings [106,109]. Moharam and Gaylord modeled a 2-D slanted fringe planar grating in the earliest 3-D RCWA model published [110]. Thin films [60,76] and thin films with gradients of refractive index [111] (at an arbitrary angle) [65,112] or porosity and stoichiometry [113] have also been modeled using RCWA, although a simpler transfer matrix method would have sufficed for any geometry that does not change in the *x* or *y* direction.

**Figure 23:**
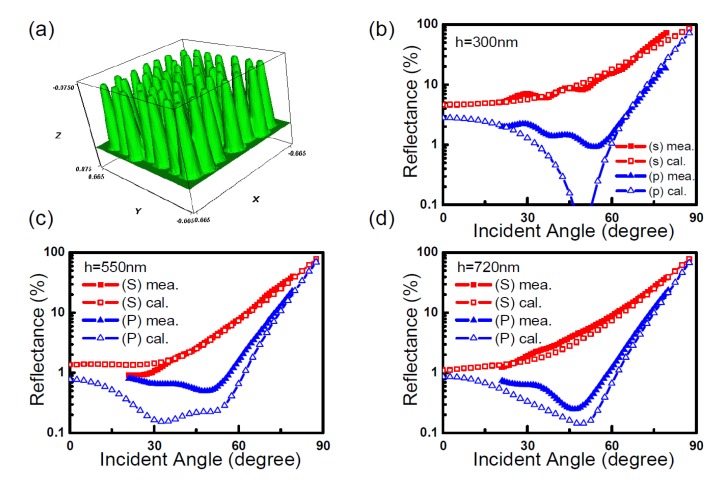
(**a**) Geometry for disordered GaN cones modeled as a 49-cone unit cell using RCWA. Measured and calculated reflectance for the disordered nanopillars shown in Figure 23 at *s* (TE) and *p* (TM) polarizations show that RCWA is more accurate in TE mode than in TM mode for pillars of (**b**) 300 nm; (**c**) 550 nm; and (**d**) 720 nm. Reprinted with permission from reference [114], Copyright 2008 Optical Society of America.

**Figure 24:**
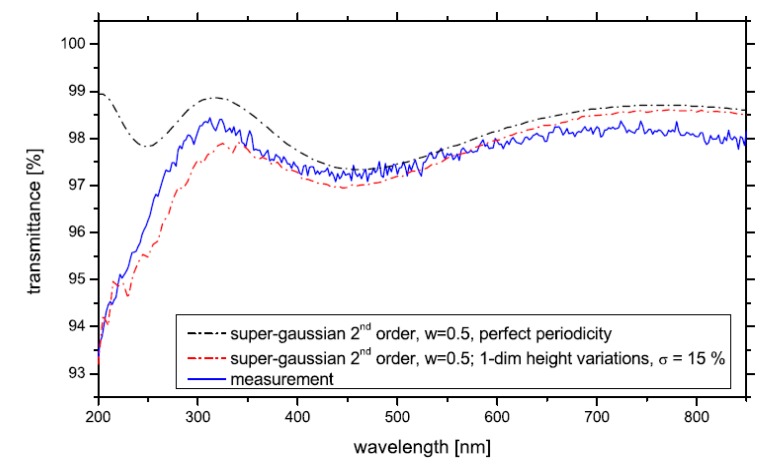
RCWA simulations accurately predict experimental transmission results by modeling the SWS as a super-Gaussian profile with one dimensional height variations with a standard deviation of 15%. Reprinted with permission from reference [114], Copyright 2010 Optical Society of America.

One of the requirements for the RCWA method is that the geometry of the model be periodic or unchanging in the *x* and *y* directions in each layer. This is a product of the Fourier series used to describe the geometry of the permittivity. However, it is possible to create an artificial disordered geometry by making the unit cell very large and having many subwavelength structures inside that unit cell. Two groups, Chiu *et al*. [114] and Lehr *et al*. [115] used this technique to simulate a disordered geometry. Chiu *et al*. [114] reported a pseudo-periodic geometry that consisted of repeating blocks of a unit cell that contained 49 cones loosely distributed in a 7 × 7 grid, but with some disorder to the exact placement of the cones (see Figure 23). Figure 23 shows the modeling and experimental reflectance results of three different heights of disordered nanopillars between 0° and 90° angle of incidence [114]. This study reported the results from the TE and TM plane waves separately. It is clear from these three graphs that RCWA models the TE polarization more accurately than TM. Lehr *et al*. [115] were able to match experimental measurements for transmittance nearly perfectly after adding some random height variation into their 2nd order super-Gaussian SWS (see Figure 24).

Several alterations to the RCWA method have been made to improve the versatility of the method. When modeling one-dimensional gratings using RCWA there are significant differences between TE and TM polarized light (see Figure 8). It has been found that TM polarized light is less accurate to model and produces convergence problems in some cases, especially in metallic gratings [108,114,116]. Lalanne and Morris [117], Granet and Guizel [118], and Li [73] developed a way to combat this problem that is sometimes called the Fourier Modal Method, or FMM, which is able to handle both TE and TM polarized light. Currently FMM and RCWA are considered to be the same method [1,71].

One limitation of the RCWA method is that it does a poor job of modeling structures with very shallow slopes. This is because it requires significantly more layers to accurately describe a shallow slope than a steep slope. To overcome this problem the coordinate transfer method, also called the Chandezon method or C-method, can be used [119]. The C-method, introduced by Chandezon and Granet [120], uses a curvilinear coordinate system transfer to naturally handle shallow slopes in the RCWA method. The C-method, however, fails for steep slopes. Thus it is recommended that a hybrid of the RCWA and C-method be used to handle all ranges of geometries [119].

**Figure 25:**
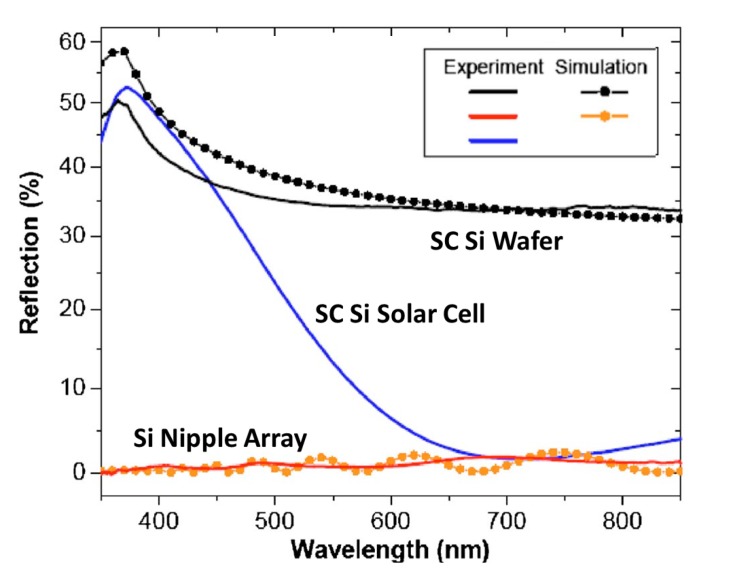
Experimental and simulated results from flat (black) and etched nipple array (red) show good agreement for the RCWA method. Blue line is an experimental value from a commercial c-Si solar cell. Reprinted with permission from reference [81], Copyright 2008 AIP Publishing.

As one of the rigorous optical modeling methods, RCWA is known to be very accurate when applied appropriately, with sufficient discretization of layers, retention of Fourier terms and calculation of diffracted orders. Figure 25 shows an example of experimental and RCWA modeling results of the reflectance of a smooth crystalline silicon wafer and a wafer with an etched nipple array for wavelengths between 350 and 850 nm [81]. As expected the RCWA simulation is decently accurate, and, in the case of the smooth wafer where the experimental result shows less reflectivity than expected, the simulation and modeling results at least follow the same trends.

**Figure 26:**
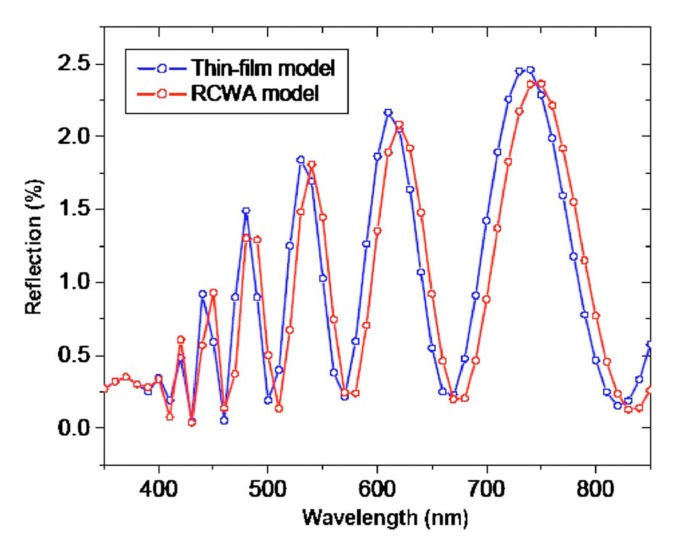
The thin film multilayer model (TMM) and RCWA show very similar results for moth eye structures with 210 nm bases and 800 nm heights. Reprinted with permission from reference [81], Copyright 2008 AIP Publishing.

RCWA results have been compared to many other methods in the literature, including ray tracing [103], the transfer matrix method [102], effective medium theory [67,72,97], and finite-difference time-domain [7,8,11]. Ray tracing is known to be ineffective for modeling sub-wavelength structures [121]. The transfer matrix method was found to agree strongly with RCWA for modeling some nipple arrays [81] (see Figure 26). However, it was found that a simple effective medium theory cannot predict some important features of moth eye reflectance [72] and that effective medium theory is only appropriate when the feature size is much smaller than the wavelengths considered, while RCWA will work at any period to wavelength ratio [67]. However, Grann *et al*. [97] demonstrated that effective medium theory and RCWA give similar results for the reflectance of Klopferstein structures. Several optical effects of ARSWS, such as the shifting low reflectance region with period, cannot be predicted with EMT and require a more rigorous model such as RCWA to model effectively [72]. Results from RCWA and FDTD have been shown to be very comparable by several authors [7,8,11].

### 5.3. Finite Element Method

The finite element method (FEM) is a frequency-based optical modeling method that, like the finite-difference time-domain method, solves for the electric and magnetic field strengths throughout the spatial computational domain. The domain is divided into finite elements, or tetrahedral meshes, and the field strengths are calculated at the vertices of the mesh. The electric and magnetic fields are represented by time-harmonic complex vectors, with the time dependency described as exp(−*i*ω*t*). The material optoelectric properties, permittivity and permeability, can be written as tensors to describe the anisotropic properties of the material. As with all frequency-based optical modeling methods, dispersion, or wavelength-dependence of permittivity, is handled simply and naturally due to the restriction of modeling one wavelength per simulation [55].

Maxwell’s equations in the harmonic regime are:

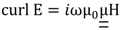
(37)

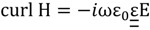
(38)

The FEM sets up these equations in matrix form as:

*A* · *x* = *b* (39)

where *A* is a square, sparse, symmetric matrix that represents the material properties at nodes, *x* is the unknown spatial components of the electric or magnetic fields, and *b* is a known vector that is determined from boundary conditions and forced excitation. This system of equations is solved either directly for small simulations or iteratively for large simulations until the model satisfies Maxwell’s Equations (37) and (38).

**Figure 27:**
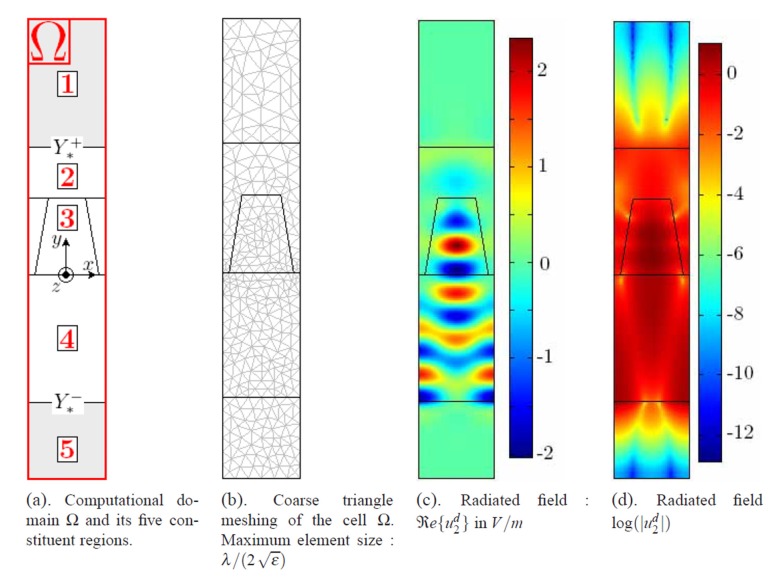
Diagram of FEM simulation, including meshing scheme. Figure Reprinted with permission from reference [123], Copyright 2007 Optical Society of America.

Many of the advantages of FEM come from the method’s meshing procedures. Meshing is the most important determinator of the accuracy of an FEM model [122]. An example computational domain, diagram of meshing, and resulting electric field strengths can be found in Figure 27. Meshing a simulation can be very computationally demanding, in some 3-D cases, can take longer than solving the simulation. However, the flexibility of the meshing available in FEM provides distinct advantages. It is possible to create a conformal mesh at boundaries between different materials, increasing the accuracy of the method and eliminating the need for staircase approximations, which are usually used in the FDTD or RCWA methods. Like any spatially-discretized modeling method, FEM requires appropriate boundary conditions, such as a perfectly matched layer, to eliminate artificial reflections at the edges of the simulation space [123]. Another advantage of FEM, unrelated to the meshing capabilities, is the possibility of multiphysics modeling; FEM optical models can be coupled to mechanical or thermal models, which is effective for modeling solar cells or temperature-dependent optical properties [55].

FEM has been used to model 2-D and 3-D random triangular gratings [124], 1-D and 2-D trapezoidal backside thin film solar cell diffraction gratings [55], and anisotropic anti-reflective gratings [123]. Two of these works reported the effects of modeling a grating in two dimensions *versus* three dimensions. Hishikawa *et al*. [124] showed that random triangles or triangular pyramids were more effective anti-reflective structures when modeled in three dimensions than in two, and that their two dimensional simulation had an accuracy of ±3%. Isabella *et al*. [55] took advantage of the multiphysics capabilities of the method to model the short circuit current, I_SC_, of an amorphous silicon thin film solar cell deposited on a diffraction grating (not an anti-reflective surface). This group found that the textured backside of the solar cell increased the I_SC_ by 25.5% and 32.5% for 1-D and 2-D structures, respectively. As FEM handles any geometry, multiphysics simulations, and anisotropy naturally, it is an appropriate method for optical simulation of thin film solar cells.

**Figure 28:**
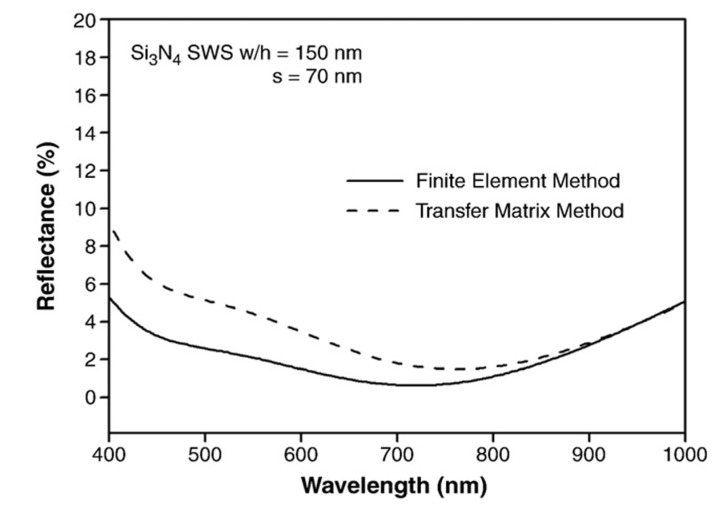
Comparison of reflectance calculations from FEM and TMM. Reprinted with permission from reference [125], Copyright 2010 Elsevier.

The finite element method is effectively based on the transfer matrix method, except that the geometry of FEM is discretized to sufficiently describe the shape and size of subwavelength structures. This eliminates the need for effective media approximations, such as are used in the transfer matrix method. Lee *et al*. [125] compare the reflectance calculated by FEM and TMM over 400 to 1000 nm wavelengths for SWS with heights of 150 nm and widths of 70 nm (see Figure 28). It is clear that the two methods give the same result for very large wavelengths, when the period to wavelength ratio is very small. However, at shorter wavelengths the assumptions behind the effective medium approximation used in TMM are no longer met, resulting in a decrease in accuracy.

FEM has been used only sparingly in the literature to model the optics of anti-reflective coatings. Most commonly the method has been used to model the optics of thin film solar cells [55,124]. Thorough information on FEM must be found from related works, not specifically anti-reflective modeling studies. Andonegui and Garcia-Adeva assess the fitness of FEM for optical modeling of photonic crystals and resonant cavities [122]. In this article they site some complaints that FEM modeling software is either inflexible or too complex for practical studies. The authors compare FEM to FDTD optical modeling; both methods handle arbitrary geometries and are computationally intensive. They find that FEM is stable, robust, rigorous, and reliable and is in some cases superior to more commonly used optical modeling methods when calculating extremely sensitive quantities. The authors noted that comparisons between FEM and FDTD can be difficult, as FEM handles only monochromatic radiation per simulation, while FDTD models a range of wavelengths naturally and some post processing is necessary to compute the Q factor (quality factor) from the FDTD method, while the Q factor is simple to compute using FEM. However, the authors found that FEM was able to accurately reproduce modeling results originally computed using FDTD.

## 6. Conclusions

This article reviews the current popular modeling methods and their results for the anti-reflective properties of sub-wavelength structures. Characteristics of FDTD, FEM, TMM, and FMM/RCWA are summarized in Table 2.

In general, these optical modeling techniques can be described by their spatial discretization techniques and their time- or frequency-based treatment of Maxwell’s equations. Spatially discretized methods (FDTD and FEM) produce field strength results for each discretized point and naturally handle arbitrary geometries. Other methods provide only reflected or transmitted efficiency, though RCWA/FMM provides the efficiency of any diffracted order of interest. Time-based methods (FDTD) are capable of inputting a range of wavelengths into one simulation. Frequency-based approaches must be solved for each wavelength, which gives them the advantage of handling dispersion naturally. TMM, though intended for thin film simulations only, can be used in conjunction with effective medium approximations to model the optics of sub-wavelength structures. The accuracy of TMM suffers, though, when structures approach wavelength sizes.

**Table 2:** Summary of features for the four main modeling methods for ARSWS.

Features	FDTD	FEM	TMM	FMM/RCWA
Geometry Restrictions	None	None	Thin Films Only	Not efficient for aperiodic surfaces
Time or Frequency Based	Time	Frequency	Frequency	Frequency
Output	Field Strengths	Field Strengths	%R/%T	%R/%T
Spatially discretized	Yes	Yes	No	No
Models dispersion naturally	No	Yes	Yes	Yes
Multiple wavelengths per simulation	Yes	No	No	No
Rigorous	Yes	Yes	Yes	Yes
Anisotropic gratings	Yes	Yes	No	Yes
Computation Speed	Slow	Meshing slow, computation fast	Fast	Medium
Source of Inaccuracies	Discretization of geometry and rounding error	Discretization of geometry and rounding error	EMT or slicing of geometry into layers	Truncation of Fourier series expansions for field values, permittivity and truncation of orders of diffracted light
Numerical convergence	Difficult for some metals, dispersion, and wavelength-sized features	Good	Good	Difficult for TM polarization
Maximum Dimensions	3D	3D	1D	3D

Commercial modeling software is available for each of these modeling methods. Most modern software enables these methods to handle simulations for which the method is not normally appropriate, such as aperiodic structures (RCWA), dispersion (FDTD), and multiple wavelengths (frequency-based methods). For the most accurate optical simulation of anti-reflective sub-wavelength structures it is best to use a variety of modeling methods to account for the disadvantages of each.

## Supplementary Materials

Supplementary File 1
