# Supplementary material for: Numerical Modeling of Sub-Wavelength Anti-Reflective Structures for Solar Module Applications
Source: Nanomaterials (Basel). 2014 Jan 29;4(1):87–128. doi: 10.3390/nano4010087 (PMC5304610; doi:10.3390/nano4010087)
Supplement: Supplementary File 1 [file nanomaterials-04-00087-s001.pdf]

Supplementary Information

Table S1. Semi-analytical effective medium approximations for 1-D and 2-D periodic structures[14].

Grating Dimension	Polarization	Order	Formula
1-D	TE	2nd	$\eta = \varepsilon_0 + \alpha^{-2} \sum_{p \neq 0} \frac{\varepsilon_{-p}\varepsilon_p}{p^2} + O(\alpha^{-4}).$
1-D	TM	2nd	$\eta = \frac{1}{a_0} + \alpha^{-2} \frac{1}{a_0^3} \sum_{p \neq 0} \frac{a_{-p} b_{p0}}{p} + O(\alpha^{-4}).$
2-D	Unpolarized	0th	$\eta_0 = \varepsilon_{0,0} - \sum_{p,q \neq 0} \sum_{m \geqslant 0, n > 0} \varepsilon_{m,n} a_{m,n}^{p,q} \varepsilon_{p,q} n.$
2-D	Unpolarized	2nd	$\eta_2 = \sum_{p,q \neq 0} \sum_{m \geqslant 0, n > 0} \varepsilon_{p,q} a_{m,n}^{p,q} c_{mn} + \sum_{p \neq 0} \frac{\varepsilon_{p,0}}{p^2 \rho^2} \left(\varepsilon_{p,0} - \sum_{\substack{(r,t) \neq (0,0) \\ u \geqslant 0, v > 0}} \varepsilon_{p-r,t} a_{r,t}^{u,v} \varepsilon_{r,t} t \right)$

Table S2. Literature summary of finite-difference time-domain modeling results for maximum anti-reflective properties.

Authors, Year	Structure Shapes	Nanostructure Material	Lattice Size (nm)	Structure Widths (nm)	Structure Heights (nm)	Λ (nm)	Results
Deinega, Valuev, Potapkin, Lozovik, 2011	Triangular-based pyramids, closest packed	$n = 1.5$	Λ	$\Lambda/3^{0.5}$	0 to 5Λ	$\Lambda/\lambda = 0.1$ to 10	Minimal reflectance occurs when the GRIN structures have diameters on the order of the wavelength and the height is large in comparison to the wavelength. For our solar spectrum a diameter around 500 nm is sufficient for $R < 1\%$
	Square-based pyramids, closest packed	$n = 1.5$	Λ	Λ	0 to 5Λ	$\Lambda/\lambda = 0.1$ to 10	
	Hexaganol-based pyramids, closest packed	$n = 1.5$	Λ	Λ	0 to 5Λ	$\Lambda/\lambda = 0.1$ to 10	
	Cones, closest packed	$n = 1.5$	Λ	Λ	0 to 5Λ	$\Lambda/\lambda = 0.1$ to 10	

Table S2. *Cont.*

Deniz, Khudiyev, Buyukserin, Bayindir, 2011	Hexaganol nanorods, tapered nanorods, thin films with nanorods on top	Hydrogen silsesquioxane	130 nm	80 nm	0–400	400–800 nm	Optimum nanorod height was 175 nm, Measured transmission lower than simulated though similar behavior with wavelength
Park, Shin, Kang, Baek, Kim, Padilla, 2011	Si substrate and cones with PS nanoislands on top	Polystyrene nanoislands and Si cones	500 nm	Cone 300 nm top, 500 nm base	Nanoislands 0, 50, 100 nm thick on top of 500 nm cone	Broadband, 300–900nm	50 nm thick nanoislands of PS decreased reflectance most
Son, Verma, Danner, Bhatia, Yang, 2011	V-shaped nanoholes	$n = 1.5$	100 nm	60 nm	300 nm	400–1200 nm	Nanostructure increases transmission at least 3% at all angles of incidence up to 70^0
Yi, Lee, Park, 2011	Conical (5^0 angle) and cylindrical nanowires	Si	1500 nm	200 and 1000 for cylinders	1500 nm	300–800 nm	Reflectivity from substrate and nanocone array were 47% and 2.4%, cylinders were 22% *R* for 200 nm wide and 29% *R* for 1000 nm wide.
Chuang, Chen, Shieh, Lin, Cheng, Liu, Yu, 2010	Cones	Si	350	245	400	1250 nm	Unique inverse polarization at Brewster angle on moth eye structures arises from TM polarized light having a higher reflectance than TE (unlike on flat surfaces). Distorted transmission plane wavefront randomizes the oscillating direction of electric dipoles in textured Si, eliminating the Brewster effect

Table S2. *Cont.*

Deinega, Valuev, Potapkin, Lozovik, 2010	Pyramids with square bases and Cones, all closest packed	$n = 1.5$	Λ	Λ	0.1Λ to 5Λ	20 to 0.1 features per wavelength	Larger feature sizes better at fixed width to height ratios, though widths above the wavelength size rely on higher order diffractions for increased transmission.
Ting, Chen, Hsu, 2010	Cones	$n = 1.54$	300,600	300,600	300,600	400–1400 nm	For same size structures antireflective properties decrease with discontinuous arrangements (incomplete SWS coverage), aspect ratio of 2 better than 1, reflectances as low as 0.46%
Ting, Chen, Hsu, 2010	pyramids		300,600	300,600	300,600	400–1400 nm	
Ting, Chen, Hsu, 2010	Composite pyramid and rounded cone		300,600	300,600	300,600	400–1400 nm	
Chou, Cheng, Chang, Ting, Hsu, Wu, Tsai, Huang, 2009	Conical ARSWS in cholesteric liquid crystal	$n = 1.54$	300 nm	150 nm	75–300	250–800 nm	Aspect ratio bigger than 0.8 reflectance is under 1%
Deinega, Konistyapina, Bogdanova, Valuev, Lozovik, Potapkin, 2009	Cones	Si	Fill factors: 0.91 0.8, 0.5, and 0.2	40 to 600	350–520	400–800 nm	As cone height increases reflectivity decreases. FDTD shows this trend continues for sizes outside applicability of EMT. Lowest reflectivity had densest packed cones, length of 500 nm, $r = 100$ nm
Ting, Chen, Chou, 2009	Pyramids	$n = 1.54$	300	300	150–600	400–800 nm	Conical and pyramidal shapes over broadband range for low *R*. Pyramids better than cones when AR is up to 0.8. Cones transmittance increases gradually with AR average is 99.6% with AR = 2, pyramids AR from 1 to 2 transmittance 99.7%.

Table S2. *Cont.*

	Rounded Cones		300	300	150–600	400–800 nm	
Tsai, 2008	Pyramidal	*n* = 1.54	150–600	150–600	150–600	250–800	Semi-spherical polymer film ARC produced in lab and shows similar reflectivity to FDTD calculations, pyramidal structure has best performance in FDTD, nearly 1/10 to 1/100 the *R* as semi spherical and discontinuous semi-sperical, respectively
	semi-spherical		150–600	150–600	150–600	250–800	
	Discontinuous semi-spherical		300–600	150–300	150–600	250–800	
Chen, Chuang, Lin, Lin, 2007	Pyramid	Si	350	350	200, 500	366	Increasing pyramid height from 200 to 500 nm reduced reflections significantly, FDTD confirms RCWA results
Yang Zhu Zhao Cao, 2004	Glass covered with polymer nanoporous film	Polymer *n* = 1.46	Unknown	N/A	5%, 10%, 15%, 20%, 30% pore ratio films	400–800 nm	transmission 99.5% between 400 and 800 nm of the 30% porous film, simulated one wavelength at a time
Feng, Zhou, Huang, 2003	Multiple layer thin films	*n* = 2.415 and *n* = 1.444	N/A	N/A	Layers 1 through 4: 135, 464, 251, and 223 nm	1450–1650 nm	Mapping established between parameter spaces of fine (FDTD) and coarse (TMM) models, focus on iterative parameter extraction and performance convergence, demonstrated by a 180 nm bandwidth four-layer ARC with $R < 10^{-4}$ using only three FDTD calculations
Ichikawa, 2002	Regular and random triangular gratings	silica	240–360 nm	240–360 nm	550 nm	400–800 nm	Randomizing has little effect on reflection, but relieves partially relieves subwavelength requirements and some fabrication constraints
Yamauchi, Mita, Aoki, Nakano, 1996	Double layer thin film AR coatings	*n*1 = 2.76, *n*2 = 1.46 on *n* = 3.564	N/A	N/A	Film thicknesses of 138 nm and 266 nm	1500–1600	Berenger's PML ABC is better than the Mur-ABC, which was used by this group in 1993

Table S3. Correlation of modeling and experimental results for finite-difference time-domain modeling.

Authors	Paper title	Nanostructure Material	Year	Structure Shape	Lattice Size (nm)	Structure Width (nm)	Structure Height (nm)	Wavelengths (nm)	Results
Chen, Chuang, Lin, Lin	Using colloidal lithography to fabricate and optimize sub-wavelength pyramidal and honeycomb structures in solar cells	Si	2007	Pyramids	350, 200	350, 200	480, 360	250–850	500 nm height much better than 300 nm in simulation, experimental results showed fabrication of pyramidal structures with less than 1.5% reflectivity
Ting, Chang, Chen, Chou	Fabrication of an AR polymer optical film with SWS using a roll-to-roll micro-replication process	PET, $n = 1.54$	2008	Conical cylinder array	400	200	350	400–700 nm	AR < 2.45% in 400–700 nm range for experimental results, similar to theoretical predictions, simulated R is 1.87%
Ting, Chen, Chou	SWS for broadband AR application	PMMA, $n = 1.54$	2009	Cones	350	350	300	400–700 nm	Ni molded lithography fabrication of conical structures in plastics. Simulation and experimental reflectances are 0.5% and 0.54% between 400 and 650 nm
Deniz, Khudiyev, Buyukserin, Bayindir	Room temperature large-area nanoimprinting for broadband biomimetic AR surfaces	Hydrogen silsesquioxane	2011	Hexaganol nanorods, tapered nanorods, thin films with nanorods on top	130	80	200–400	400–800	Optimum nanorod height was 175 nm

Table S3. *Cont.*

Park, Shin, Kang, Baek, Kim, Padilla	Broadband Optical Antireflection Enhancement by Integrating Antireflective Nanoislands with Silicon Nanoconical-Frustrum Arrays	Polystyrene nanoislands and Si cones	2011	Si substrate and cones with PS nanoislands on top	500 nm	Cone 300 nm top, 500 nm base	Nanoislands 0, 50, 100 nm thick on top of cone, cone height 500 nm	Broadband, 300–900 nm	Determined best size for cones and islands and fabricated, 50 nm thick island was best
Yi, Lee, Park	Site-specific design of cone-shaped Si NWs by exploiting nanoscale surface diffusion for optimal photoabsorption	Si	2011	Conical (5^0 angle) and cylindrical nanowires	1500 nm	200 and 1000 for cylinders	1500 nm		Good agreement with experimental, sim results for substrate and nanocone array were 44% and 2.7%, nanocones absorbed over 96% of visible range, cylinders were 22% *R* for 200 nm wide and 29% *R* for 1000 nm wide, cylinders had 49%–64% optical absorption, not as good as cones
Son, Verma, Danner, Bhatia, Yang	Enhancement of optical transmission with random nanohole structures	$n = 1.5$	2011	V-shaped nanoholes	100 nm	60 nm	300 nm	400–1200 nm, 0 to 70 degrees AOI	solar cell efficiency increased from 10.47% to 11.2%

© 2014 by the authors; licensee MDPI, Basel, Switzerland. This article is an open access article distributed under the terms and conditions of the Creative Commons Attribution license (http://creativecommons.org/licenses/by/3.0/).
